# Substrate conformational dynamics facilitate structure-specific recognition of gapped DNA by DNA polymerase

**DOI:** 10.1093/nar/gkz797

**Published:** 2019-09-23

**Authors:** Timothy D Craggs, Marko Sustarsic, Anne Plochowietz, Majid Mosayebi, Hendrik Kaju, Andrew Cuthbert, Johannes Hohlbein, Laura Domicevica, Philip C Biggin, Jonathan P K Doye, Achillefs N Kapanidis

**Affiliations:** 1 Biological Physics Research Group, Clarendon Laboratory, Department of Physics, University of Oxford, Oxford OX1 3PU, UK; 2 Physical and Theoretical Chemistry Laboratory, Department of Chemistry, University of Oxford, South Parks Road, Oxford OX1 3QZ, UK; 3 School of Physics, Institute for Research in Fundamental Sciences (IPM), Tehran 19538-33511, Iran; 4 Laboratory of Biophysics, Wageningen University & Research, Wageningen 6708 WE, The Netherlands; 5 Microspectroscopy Research Facility Wageningen, Wageningen University & Research, Wageningen 6708 WE, The Netherlands; 6 Department of Biochemistry, University of Oxford, Oxford OX1 3QU, UK

## Abstract

DNA-binding proteins utilise different recognition mechanisms to locate their DNA targets; some proteins recognise specific DNA sequences, while others interact with specific DNA structures. While sequence-specific DNA binding has been studied extensively, structure-specific recognition mechanisms remain unclear. Here, we study structure-specific DNA recognition by examining the structure and dynamics of DNA polymerase I Klenow Fragment (Pol) substrates both alone and in DNA–Pol complexes. Using a docking approach based on a network of 73 distances collected using single-molecule FRET, we determined a novel solution structure of the single-nucleotide-gapped DNA–Pol binary complex. The structure resembled existing crystal structures with regards to the downstream primer-template DNA substrate, and revealed a previously unobserved sharp bend (∼120°) in the DNA substrate; this pronounced bend was present in living cells. MD simulations and single-molecule assays also revealed that 4–5 nt of downstream gap-proximal DNA are unwound in the binary complex. Further, experiments and coarse-grained modelling showed the substrate alone frequently adopts bent conformations with 1–2 nt fraying around the gap, suggesting a mechanism wherein Pol recognises a pre-bent, partially-melted conformation of gapped DNA. We propose a general mechanism for substrate recognition by structure-specific enzymes driven by protein sensing of the conformational dynamics of their DNA substrates.

## INTRODUCTION

Protein machines functioning on chromosomes and plasmids utilize different mechanisms to locate their targets on DNA. Sequence-specific DNA-binding proteins, such as restriction enzymes and transcription factors, recognize a particular nucleotide sequence via a combination of direct and indirect readouts (1). Whereas direct readout involves specific interactions between the DNA bases and protein amino acid side chains, indirect readout senses sequence-dependent structural and mechanical features, such as major or minor groove width, and conformational flexibility (2). In contrast, structure-specific proteins have no sequence specificity; instead, they interact with particular DNA structures (e.g. gapped duplexes, and 5′ or 3′ overhangs). While sequence-specific DNA binding mechanisms have been studied extensively, structure-specific mechanisms remain unclear.

Many enzymes involved in DNA repair and replication are necessarily structure-specific, and have been shown to interact with bent DNA substrates; examples include Flap endonuclease 1 (3–5), DNA Polymerase β (6), XPF (7) and MutS (8,9). Although catalytic reasons for substrate distortion have been suggested for individual systems, it is unclear whether the structure and dynamics of bent states serve as general recognition signals for binding or substrate selectivity. A key related question is whether these proteins induce DNA bending upon binding (via an ‘induced fit’ mechanism) or they recognise a pre-bent state adopted by the DNA prior to protein binding (a ‘conformational selection’ mechanism), or a combination of both.

*Escherichia coli* DNA polymerase I is a structure-specific protein responsible for Okazaki fragment processing in lagging-strand DNA replication, as well as for DNA synthesis during DNA repair. In both roles, the polymerase recognizes and binds to a gapped DNA substrate and polymerizes across the gap. After gap filling, strand-displacement synthesis may follow; this is important for Okazaki fragment processing, as the polymerase continues to synthesize DNA whilst displacing an RNA primer, which is subsequently excised (by the 5′-nuclease domain of the protein). In this study, we used the Klenow Fragment of DNA Polymerase I (hereafter called Pol) which harbours the gap-filling and strand-displacement DNA polymerisation activities, but lacks the 5′-nuclease domain, allowing us to focus on the initial binding interaction in the polymerase active site.

Attempts to understand the DNA binding and recognition mechanism for Pol are complicated by the absence of crystal structures of DNA–Pol binary complexes containing downstream duplex DNA, by the heterogeneity of Pol-DNA complexes (10,11), and by the conformational mobility of the free DNA substrate. As a result, there are many open questions regarding the mechanisms of strand-displacement DNA synthesis and substrate recognition.

We investigated the mechanism of structure-specific recognition by Pol via a combination of single-molecule Förster resonance energy transfer (smFRET) and molecular modelling. The single-molecule nature of our work addressed the issues of conformational and compositional heterogeneity, and led to a FRET-restrained solution structure of the binary complex, Pol bound to 1-nt gapped DNA. This structure revealed a substantial bend in the DNA substrate (supported by complementary FRET experiments in living bacteria), and provided insight into protein and DNA structural features crucial for strand-displacement synthesis. The structure also served as the starting point for atomistic molecular dynamics simulations, which revealed the dynamic nature of the binary complex and the specific interactions between the protein and DNA. Experimental smFRET measurements and coarse-grained modelling allowed us to characterize the conformational ensemble of the free substrate and propose a mechanism for substrate recognition and binding by DNA polymerase I, which is likely to apply to many other structure-specific DNA-binding proteins.

## MATERIALS AND METHODS

### Protein Expression, purification and labelling

DNA polymerase I Klenow Fragment (Pol) variants were expressed from an N-terminal-His6, D424A construct and purified using Ni-NTA affinity chromatography as described ((10) and Supplementary Methods). The D424A mutation inhibits the proof-reading exonuclease activity.

Pol variants containing a single cysteine (C907+, C907S/K550C and C907S/L744C) were labelled using a two-fold excess of the maleimide derivative of Cy3B (GE Healthcare) according to the manufacture's protocols and as described previously (10). Labelled proteins were purified on a heparin column, yielding labelling efficiencies of ∼80 % as determined by UV-Vis absorbance.

### DNA labelling and annealing

DNA oligonucleotides (oligos; Supplementary Table S3) were purchased from IBA GmbH, and labelled with NHS-ester derivatives of Cy3B (GE Healthcare) or Atto647N (ATTO-TEC) via dT-C6-amino linkers at selected positions according to the manufacturers’ protocols. Labelled oligos were purified by 20% polyacrylamide gel electrophoresis. Gapped-DNA substrates were assembled by annealing three single-stranded oligos (Supplementary Table S3).

### *In vitro* single-molecule FRET measurements

Single-molecule FRET measurements were performed at room temperature using a home-built confocal microscope with 20 kHz alternating-laser excitation between a 532-nm (Samba, Cobolt, operated at 240 μW) and a 638-nm laser (Cube, Coherent, operated at 60 μW), coupled to a 60×, 1.35 numerical aperture (NA), UPLSAPO 60XO objective (Olympus) as previously described (11). For DNA–DNA measurements, labelled DNA was present at <100 pM and unlabelled Pol (when present) at 3 nM concentration. For Pol-DNA measurements, both Pol and DNA were present at 100 pM concentration. Measurements were taken in ‘Pol buffer’, consisting of 40 mM 4-(2-hydroxyethyl)-1-piperazineethanesulfonic acid (HEPES)–NaOH, pH 7.3, 10 mM MgCl_2_, 1 mM DTT, 100 μg ml^−1^ bovine serum albumin, 5% (v/v) glycerol, 1 mM mercaptoethylamine. Photon streams in DD, DA and AA channels were recorded and processed using custom-written software (LabVIEW). Bursts were filtered for the correct labelling stoichiometry (12), and accurate FRET efficiencies were calculated as described ((13) and Supplementary Methods). Distances were calculated from the FRET efficiencies, using experimentally determined Förster radii and donor quantum yields (Supplementary Table S4).

### In vivo single-molecule FRET measurements

We internalized DNAs into electro-competent DH5α *E. coli* cells (Invitrogen) using electroporation (14). Cells were recovered, washed and transferred onto agarose-M9 pads and imaged under 532 nm continuous illumination at 50 Hz using an Olympus IX71 inverted microscope in highly inclined thin illumination mode (15). The cellular fluorescence was spectrally separated into Donor and FRET fluorescence channel and directed onto an Andor EMCCD camera. Data was analyzed using custom-written MATLAB scripts. PSFs were localized in the Donor and FRET channel in each movie frame. Localized PSFs in the FRET channel were linked to trajectories if they appeared in 5 consecutive frames within a window of 7 pixels (∼ 0.69 μm). The donor-channel was mapped onto the FRET-channel and FRET efficiencies were calculated from co-localized PSFs (see Supplementary Methods).

### All-atom molecular dynamics simulations

Protein simulations were based on the 4BDP structure (16). For DNA-only simulations, DNA models were generated using the 3D-DART server (17). Simulations were run using either the Amber ff99sb force field (18) with modified nucleic acid parameters (parmbsc0; (19,20)) or the Amber ff99sb-ILDN force-field with modifications as described in the Supplementary Methods. MD was performed with Gromacs 4.6 (21) in a triclinic box, with a minimum 10-Å solvent edge, in the presence of 10 mM MgCl_2_ and explicit water. After equilibration, unrestrained production was run for 100 ns, with the temperature of 298 K and the pressure of 1 bar maintained by the V-rescale thermostat and a Parrinello-Rahman barostat (22). Long-range electrostatic interactions were accounted for by the Particle-Mesh Ewald method (23) and bonds were constrained with the LINCS algorithm, enabling a time step of 2 fs. Repeat simulations were carried out with different initial atom velocities each time.

In the case of full-length DNA-only simulations, the production runs were 20 ns. In the case of high-temperature DNA simulations carried out as part of model preparation, the conditions were the same as for the complex simulations except that the temperature during the equilibration and production runs was 400 K, and the production times were 2 ns. All DNA heavy atoms were position-restrained during these production runs, except for the 6 base pairs in the protein-proximal, downstream part of the DNA, which were unpaired in the starting configuration.

Further details of the computational aspects of this work (rigid-body docking, all-atom and coarse-grained molecular dynamics simulations, and conversion of FRET to distance) alongside full derivations of the multistate equilibrium model and the corrections for accurate FRET efficiencies, and quFRET analysis procedures, are available in the Supplementary Methods. Full atomic coordinates (pdb format) of a representative snap-shot of the binary complex structure, and movies from the simulations are available as supplementary information.

## RESULTS

### Structural analysis of multiple species in dynamic equilibrium

To analyse the structure of the binary complex of Pol with a 1-nt gapped DNA in solution, we determined numerous DNA–DNA and protein–DNA distance restraints within freely diffusing Pol-DNA complexes using single-molecule confocal fluorescence microscopy combined with alternating-laser excitation (24–26). This approach was recently validated in a multi-lab bench marking study (13).

**Figure 1.**
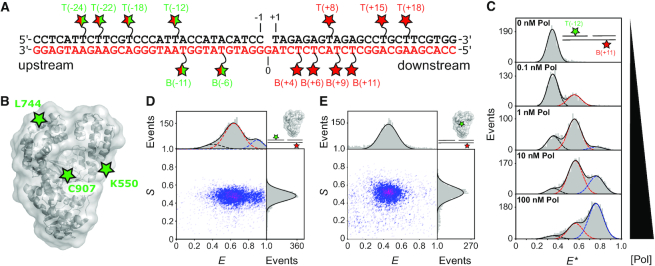
Measuring distances within single polymerase–DNA complexes in heterogeneous mixtures with dynamic species. (**A**) Schematic of a 1-nt gapped DNA substrate showing the template (red lettering) and non-template strands (black). Red stars represent acceptor labelled dT bases. Split red/green stars indicate positions labelled with donor for DNA–DNA FRET, or acceptor for DNA–protein FRET. (**B**) DNA Polymerase I (Klenow Fragment; Pol) structural schematic (grey – pdb 1KLN) and donor labelling positions (green stars). (**C**) Apparent FRET histograms for the doubly-labelled substrate T(–12) B(+11) at increasing concentrations of Pol. The data (grey bars) were fitted with up to three Gaussians (black, red and blue dashed lines), yielding apparent FRET efficiencies, E* of 0.35, 0.55 and 0.75. (**D**) Corrected ES histogram for a DNA–DNA FRET measurement (here, for T(–12)B(+11) in the presence of 3 nM Pol). Data (grey bars) were fitted by the sum of three Gaussians (solid black lines) centered on *E* = 0.41 (black dash), *E* = 0.63 (red dash) and *E* = 0.90 (blue dash) respectively. (**E**) Corrected ES histogram for a protein–DNA FRET measurement (here, for C907-Cy3B B6-Atto647N). Data were fitted with a single Gaussian function, centered on E = 0.48. See also Supplementary Figure S1.

We measured DNA–DNA distances between labelled sites in the upstream and downstream duplex regions of gapped DNA containing a 3′-dideoxy nucleotide (to prevent any chemistry occurring; Figure 1A). We also measured DNA–protein distances between a FRET donor dye attached to one of three Pol residues (K550C, L744C, C907; Figure 1B) and a FRET acceptor dye attached to one of 13 labelling sites on gapped DNA. Pol activities were not significantly affected by the dye presence (10,27).

We determined the Pol concentration required to form binary complexes by monitoring FRET between upstream and downstream sites on the DNA at increasing Pol concentrations. Three distinct FRET states were observed during the titration, indicating three different conformations of the gapped DNA (Figure 1C). A low-FRET state corresponding to free DNA; a high-FRET state corresponding to a (Pol)_2_–DNA ternary complex; and a mid-FRET state corresponding to two distinct species, the Pol-DNA binary complex, and a (Pol)_2_-DNA ternary complex, with indistinguishable FRET signals (see *Methods;*Supplementary Figure S1A-B). The increased FRET for the Pol-DNA binary complex versus free DNA suggested substantial DNA bending in the binary complex. Using global fitting (Materials and Methods), we recovered dissociation constants of *K*_D1_ = 360 ± 60 pM for the binary complex, and *K*_D2_ = 9 ± 4 nM for formation of the mid-FRET (Pol)_2_–DNA dimer species (Supplementary Figure S1B), consistent with previous observations of Pol dimers (28–30).

To characterize the structure of the DNA in the binary complex, we set the Pol concentration to 1 nM thereby maximising the population of the binary complex (Figure 1D), and measured 34 DNA–DNA FRET distances (Supplementary Table S1, and Supplementary Figure S1). We also obtained 39 protein–DNA distance restraints for the binary complex, measuring FRET between donor-labelled Pol and acceptor-labelled DNA (Supplementary Table S1 and Figure 1E). Single-molecule confocal measurements are generally limited to a maximum concentration of fluorescent species of ∼100 pM, however, because of its low dissociation constant, a detectable quantity of the binary complex was present even at 100 pM dye-labelled Pol (Figure 1E), for all DNA and Pol labelling positions tested, suggesting the labels have little effect on the DNA–Pol interaction.

### The DNA substrate is bent by 120° in the Pol-DNA binary structure

**Figure 2.**
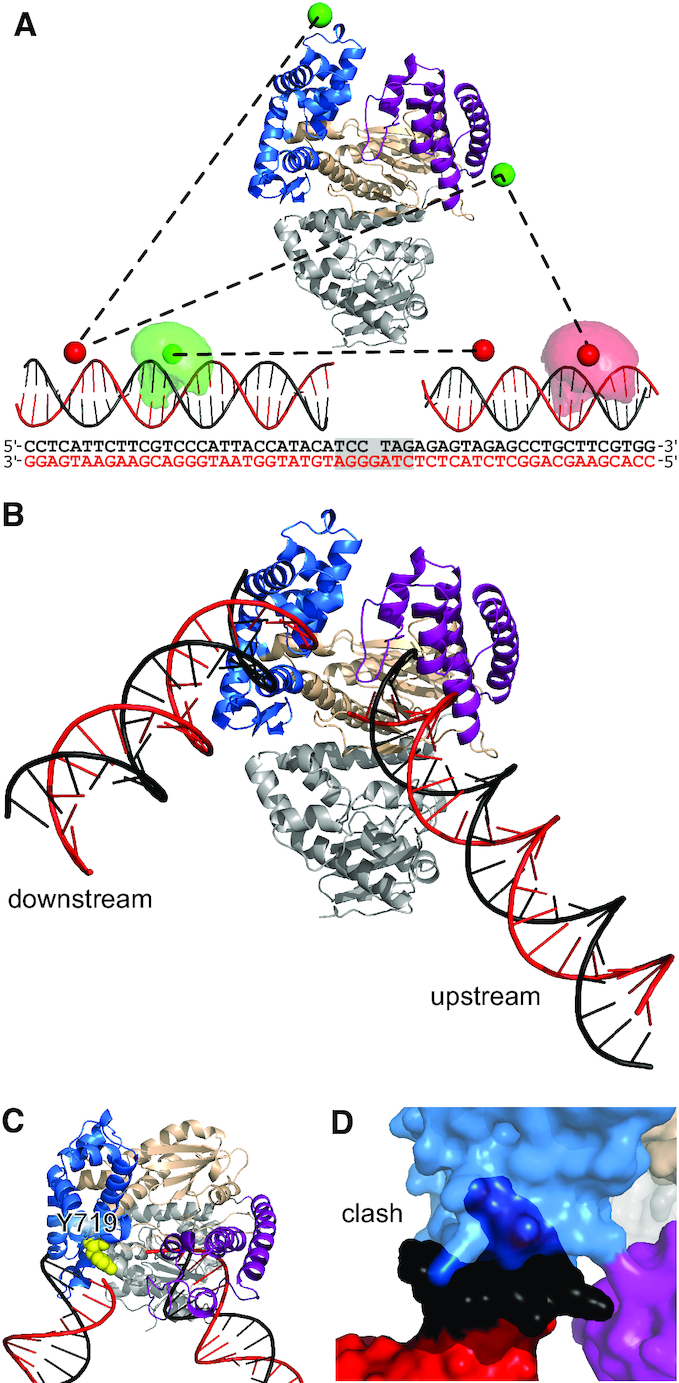
Pol-DNA binary structure from rigid-body docking. (**A**) Pol structure showing the fingers (blue), thumb (purple) and palm (wheat) subdomains and the proof-reading exonuclease domain (grey). Example DNA–DNA and protein DNA-distances (black dashed lines) are shown between mean dye positions (green and red spheres). Example accessible volumes of a donor (pale green cloud) and an acceptor (pale red cloud) dye are also shown, along with the full sequences of the docked DNAs; the shaded region indicating the DNA not used for the docking. (**B**) Results of the rigid-body docking: template DNA (red), non-template DNA (black), subdomains coloured as in (A). (**C**) Position of Y719 relative to downstream DNA. (**D**) Clash between full-length downstream DNA and the fingers subdomain (cyan). See also Supplementary Figure S2 and Supplementary Table S1.

To obtain structural models of the Pol-DNA complex, we used our 73 distance restraints to perform rigid-body docking using Pol and two shortened DNA helices representing the upstream and downstream DNA (Figure 2A). We generated 32 refined structures and ranked them according to their fit to the measured distances (see Materials and Methods). A single model (Figure 2B) emerged with a significantly better fit than the other structures (Supplementary Figure S2A). In this model, the position of the upstream DNA agreed very well with the position of a DNA fragment in a crystal structure of a Pol-DNA binary complex (31), (RMSD = 2.9 Å; Supplementary Figure S2B), demonstrating the accuracy of our structural model. To test the robustness of our model, we generated 100 ‘bootstrapped’ structures by randomly perturbing the 73 distance restraints in proportion to their experimental errors, repeating the docking calculations, and calculating the RMSD for each DNA backbone phosphate atom across all bootstrapped structures (average RMSD = 3.8 Å, Supplementary Figure S2E).

Having established the accuracy and precision of our model, we inspected it for insights into the DNA-binding and strand-displacement mechanisms. The most striking feature was the significant kink in the DNA substrate, a ∼120° bend compared to straight duplex (Figure 2B). Further, the downstream DNA is positioned close to the fingers subdomain (Figure 2C), with the helical axis aligned with Y719 (*Bst* numbering used as default; this corresponds to residue F771 in *E. coli*). Substitution of this residue with alanine was previously shown to significantly impair strand displacement synthesis by Pol (32). We rebuilt the downstream DNA in our docked model to its full length, by modelling the previously deleted base pairs proximal to the gap as B-form duplex. However, this resulted in a clash between the additional DNA and the Pol fingers (Figure 2D), indicating that DNA immediately downstream of the gap may be partially melted in the binary complex.

We also used 21 FRET restraints (Supplementary Table S1) to obtain the relative orientation of the upstream and downstream DNA in the high-FRET (Pol)_2_–DNA ternary complex and generated a low-resolution model for this complex in which the DNA was more severely bent (by ∼140°; Supplementary Figure S2F; RMSD = 12 Å, Supplementary Figure S2G).

### All-atom MD simulations give structural insights into the strand displacement mechanism

To probe the exact position of DNA in the binary complex, its dynamics and any specific contacts with Pol, we carried out all-atom MD simulations. We generated five different starting models by combining the DNA from our FRET-restrained structure with the short DNA fragment present in the 4BDP Bst X-ray structure (16) (see *Methods* and Supplementary Figure S3B), and performed two unconstrained 100-ns simulations from each model.

**Figure 3.**
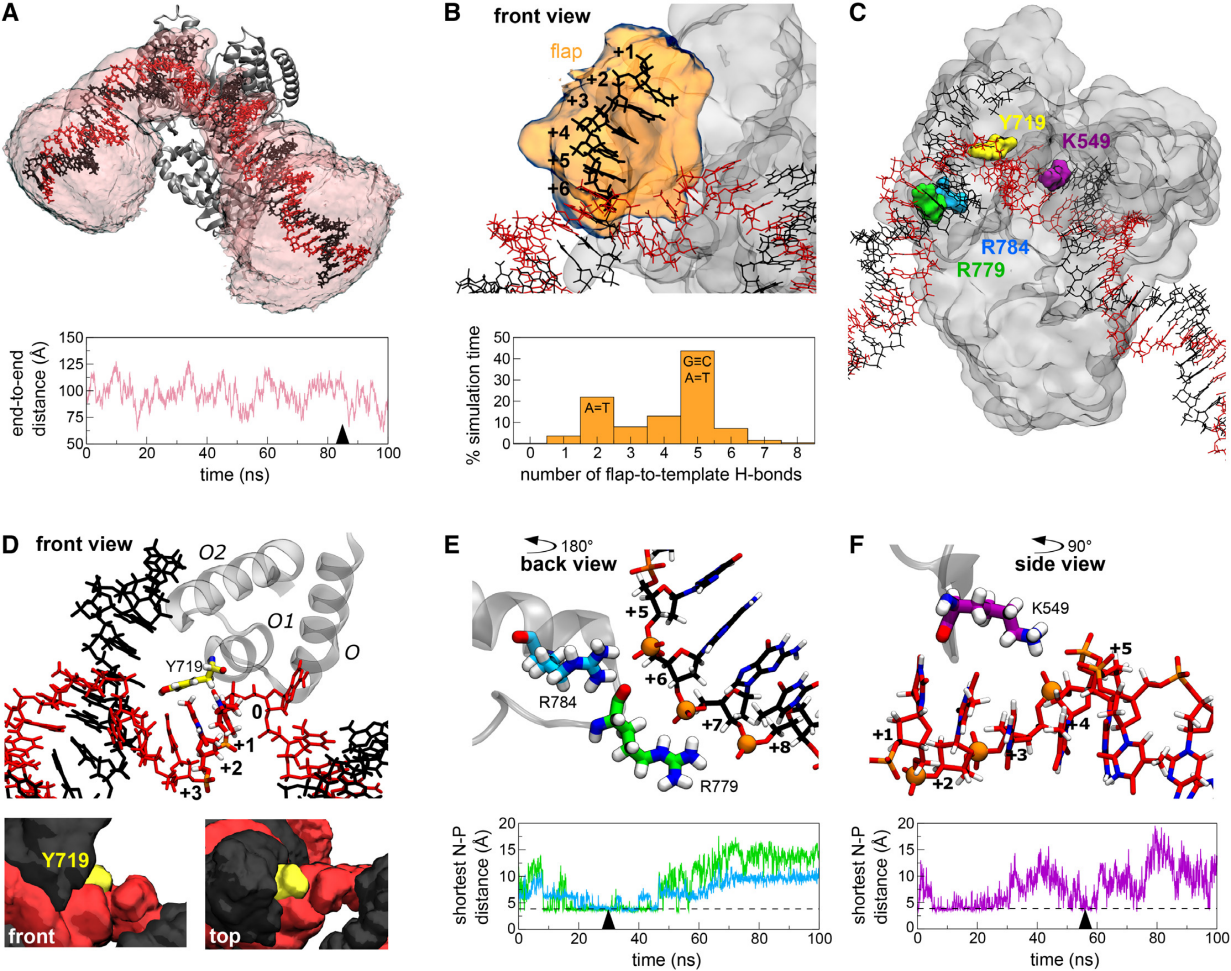
Binary complex structure and dynamics. (**A**) Representative snapshot of the DNA–Pol binary complex from a 100-ns MD simulation, showing the volume accessed by the DNA over the simulation (pale pink). The plot shows the DNA end-to-end distance fluctuations over the same simulation, with the ends taken as the terminal non-hydrogen atoms of the template strand. The time point corresponding to the snapshot is indicated with an arrowhead. (**B**) Representative snapshot of the conformation of the 6-nt non-template flap, with its volumetric map during a 100-ns simulation (pale orange). The plot shows the frequency of the number of hydrogen bonds formed between the flap and the template strand of downstream DNA during the entire 1-μs (10 × 100 ns) simulation time. (**C**) Overview of Pol residues involved in strand separation or interactions with downstream DNA. See also panels (D) to (F). (**D**) Involvement of Y719 in strand separation of downstream DNA. Top - a representative snapshot of the position of Y719 relative to the template DNA strand. The three DNA residues positioned closest to Y719 during the time course of the simulation are highlighted in CPK colouring. The position of the three-helix bundle is shown for reference; the rest of the protein is omitted for clarity. Lower panels, two different views of the volumetric maps of Y719 (yellow), template (red) and non-template DNA strands (black) during a 100-ns simulation. (**E**) A representative snapshot of the interactions between R779 (green) and R784 (cyan) with phosphate groups (orange spheres) in the non-template strand of downstream DNA. The plot shows the minimum distance between the side-chain nitrogen atoms of R779 (green) or R784 (cyan) to any phosphorous atom in the non-template strand of downstream DNA during a 100 ns simulation. Arrowhead denotes the time point corresponding to the snapshot, and dashed lines indicate the distance corresponding to an interaction. (**F**) A representative snapshot of the interaction between residue K549 (purple) with phosphate groups in the template strand of downstream DNA. See also Supplementary Figure S3, Movie M1 and PBD File P1.

Whereas the DNA fragment present in the X-ray structure remained stably bound to Pol (RMSD 2.8 ± 0.8 Å), the upstream and downstream segments flanking this DNA were much more mobile (RMSD 11.1 ± 4.4 Å and 19.2 ± 8.8 Å, respectively; Supplementary Figure S3C), with the end-to-end DNA distance ranging from 24 to 144 Å (Figure 3A and Supplementary Figure S3D). The first six nucleotides of the downstream, non-template DNA (nucleotides T(+1) to T(+6), termed the non-template flap) also displayed appreciable dynamics (RMSD 8.7 ± 3.7 Å, Figure 3B and Supplementary Figure S3E), and did not dock in a particular conformation. To study the extent of DNA melting in this region, we counted the hydrogen bonds formed between the six nucleotides of the non-template flap and the template strand: for most of the simulation time, 2 or 5 hydrogen bonds were present (Figure 3B), corresponding either to a single A–T or to an A–T plus a G–C pair, respectively, consistent with base-pairing of the two nucleotides at the base of the flap. Hence, in most conformations, four or five nucleotides of the flap were melted. Contacts between the flap and Pol were transient and diverse in terms of the residues involved; the most consistent interactions were sequence-unspecific, being formed between DNA phosphates and positively charged residues (mainly R729 and K730).

Many of the protein–DNA interactions in the active site (Y714, S717, Y719 and R789, all contacting the template strand) were similar to those observed in the X-ray structure (16). Further, in our simulations, the conserved three-helix bundle (O, O1 and O2 helices in the fingers subdomain), and especially residue Y719 (F771 in *E. coli*) were consistently positioned between the downstream template and non-template strands (Figure 3D); Y719 was typically positioned perpendicular to bases B(+1) and B(+2) of the template strand (Figure 3D, upper panel), and occasionally stacked against them (Supplementary Figure S3F). The position of Y719 is consistent with a previously suggested mechanism in which Y719 acts as a ‘wedge’, separating the non-template strand from its template counterpart (32). Despite the intrinsic dynamics of the non-template strand, the stable positioning of Y719 against the template strand likely prevents re-pairing during catalysis.

Finally, we observed interactions between downstream DNA and the polymerase, which consistently involved positively charged residues on the Pol surface and the negatively charged phosphate groups of the DNA backbone. These interactions occurred in two regions: the first involved R779 (S831) and R784 (R836) that contact the duplex region of downstream DNA (Figure 3E), and the second featured K549 (K601) of the thumb region interacting with the unpaired template strand (Figure 3F). Whilst any individual nitrogen–phosphate interaction was transient, each residue contacted up to 6 phosphate groups, resulting in Pol–downstream DNA interactions persisting for most of the simulation time. The dynamic nature of these interactions likely reflects the need for rapid Pol movement along its DNA substrate during DNA synthesis.

### Downstream DNA is melted in the DNA–Pol binary complex

To study the melting of the downstream non-template strand predicted by both our docked binary complex model (Figure 2D) and MD simulations (Figure 3B), we used quenchable FRET (quFRET), a single-molecule assay able to detect local DNA unwinding (33–35). In quFRET, when the donor (Cy3B) and acceptor (Atto647N) are in close proximity (<2 nm), their emission is quenched, yielding only few events with intermediate stoichiometry (0.4 < *S* < 0.8) (see Supplementary Methods). Upon local DNA melting, the two dyes move further apart and the quenching is reduced, leading to a large increase in both the number, and proportion of events with intermediate stoichiometry (mostly occurring at high FRET efficiencies, as the inter-dye distance remains short).

**Figure 4.**
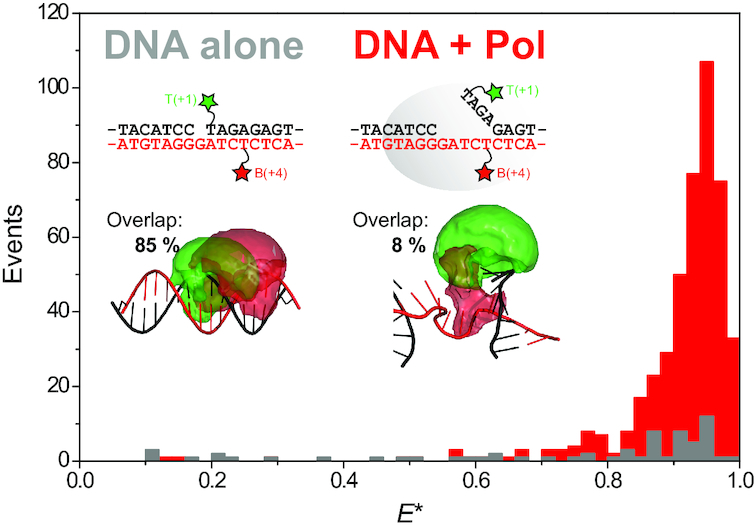
Downstream DNA is melted in the binary complex. Quenchable FRET data are plotted, showing the number of events with mid-stoichiometry (0.4 < *S* < 0.8) versus the apparent FRET efficiency, for DNA substrate T(+1)B(+4) alone (grey bars) and in the presence of 3 nM Pol (red bars). Inset: Schematics of the labeling positions and DNA structures for the unbound (left; B-DNA) and bound conformations (right; snapshot from MD simulations, atomic coordinates provided as SI), and the related accessible volumes of the donor (green) and acceptor (red) dyes, quoting the percentage overlap between them (see main text). See also Supplementary Figure S4.

We studied a 1-nt gapped DNA substrate labelled with donor and acceptor dyes at positions T(+1) and B(+4), respectively. In the absence of Pol, the dyes are in very close proximity; as a result, we detected few intermediate-S events (Figure 4), comprising only ∼25% of all acceptor-containing molecules (Supplementary Figure S4A). On addition of Pol, we observed a ∼4.5-fold increase in the number of such events per measurement, with a peak at high FRET (E* > 0.9; Figure 4), now comprising ∼75% of all acceptor-containing molecules (Supplementary Figure S4B). These results demonstrate an increase in dye separation and reduced quenching, consistent with the presence of local melting at the 5′-end of the downstream non-template strand in the binary complex.

To monitor the extent of melting along the downstream DNA, we tested a substrate with donor and acceptor dyes at B(+9) and T(+8) respectively. For these labelling positions, we observed similar quenching in both the absence and presence of Pol (Supplementary Figure S4D–F), implying that this DNA site remains base-paired in the binary complex. This suggests the maximum number of melted base-pairs in the binary complex is seven, consistent with the 4–5 observed in our MD simulations.

To relate the observed quenching changes to DNA rearrangements upon Pol binding, we analysed the accessible volumes (AVs) of the dyes in both bound and unbound conformations. Assuming a B-DNA duplex conformation for the unbound substrate, 85% of the donor AV overlapped with the acceptor AV (Figure 4, inset); this was reduced to 8% in representative MD snapshots of the binary complex in which 4–5 nt are melted. Additionally, no quenching was observed for substrates with dye labelling positions with 0% AV overlap (Supplementary Figure S4G–I). Hence, the observed reduction of quenching in the bound state correlated well with the change in AV overlap. Taken together, our quFRET results strongly support the hypothesis that the downstream duplex DNA is partially unpaired in the binary complex.

### The free 1-nt gapped DNA substrate adopts bent and frayed states

To examine to what extent DNA bending and downstream melting were present in the free substrate, and to establish whether Pol recognizes such structural features via conformational selection, or induces them upon binding, we studied the structure and dynamics of the 1-nt gapped DNA substrate in the absence of Pol.

**Figure 5.**
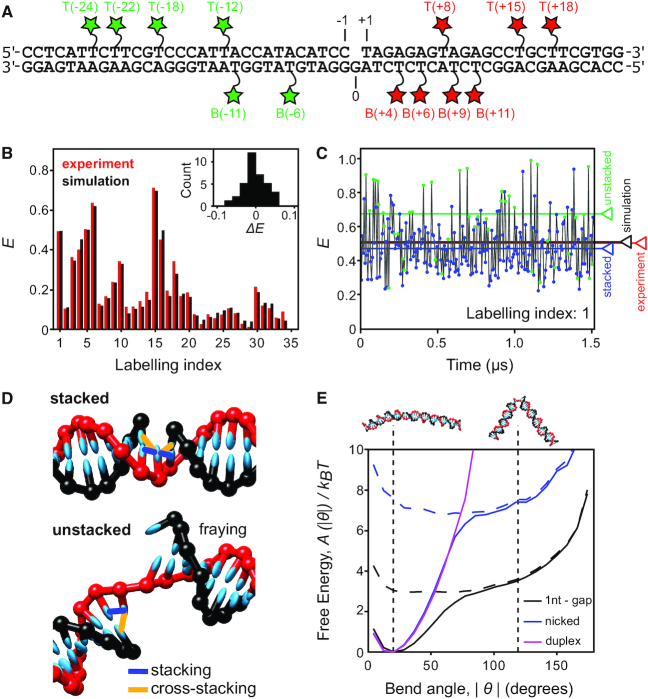
Gapped DNA substrate adopts highly bent conformations due to base unstacking. (**A**) Schematic of the 1-nt gapped DNA substrate. Green and red stars indicate donor and acceptor labelled dT bases, respectively. (**B**) Comparison of experimental (red) and simulated (black) corrected FRET values for 34 FRET measurements in the 1-nt gapped substrate (Labelling index listed in Supplementary Table S2). Inset: residual histogram, Δ*E* = *E*_model_ – *E*_measured._ (**C**) Simulated FRET time trace for substrate T(+8)B(–11), labelling index 1. Stacked (blue circles) and unstacked (green circles) states are identified. The average FRET efficiency across the entire simulation is shown for each state individually (blue and green lines), for the sum of both states (black line) and for the experimentally determined FRET value (red line). (**D**) Typical snapshots of the stacked and unstacked states indicating the stacking (blue) and cross-stacking (orange) interactions present. (**E**) Free-energy profiles as a function of the bend angle (θ = 0 corresponds to a straight duplex) for 1nt-gap (black), nicked (blue) and duplex (magenta) DNAs. The total free energy (solid lines) and the contribution due to unstacked states alone (broken lines) are shown. The vertical dotted lines and cartoons correspond to the most stable bend angle (20°) and the bend angle observed in the binary complex structure (120°). See also Supplementary Figure S5, Supplementary Table S2 and Movie M2.

We used smFRET to collect 34 DNA–DNA distances within the free DNA (Figure 5A-B and Supplementary Table S2). Corrected ‘ES’ histograms showed a single FRET peak, consistent with either a single DNA conformation or rapid (sub-millisecond) conformational averaging (Supplementary Figure S5A-C). Rigid-body docking of the two duplex portions of the substrate failed to produce a unique structural model; instead, five structures of the gapped-DNA emerged, with angles between the duplex arms spanning from 8° to 25° (Supplementary Figure S5D). This approach assumed a single static structure was responsible for the experimental FRET distances; however, the substrate is expected to be highly dynamic (36,37). To take into account these dynamics, we conducted coarse-grained molecular modelling on the gapped-DNA substrate using the oxDNA model, which allows rapid and efficient conformational sampling, and has been shown to describe well the structural, thermodynamic and dynamic properties of many DNA systems (Supplementary Figure S5E) (38–41).

Using an adapted AV approach (see Materials and Methods and Supplementary Figure S5G) (42,43), we calculated the FRET efficiency arising from each dye pair at regular simulation intervals (Figure 5C). Transitions between different configurations were rapid (sub-microsecond) and much faster than the temporal resolution of the smFRET experiments (∼1 ms; see Materials and Methods). Therefore, the average FRET efficiencies from the simulations are expected to agree with those measured experimentally. Indeed, we see excellent agreement between the experimental and modelled FRET efficiencies across all 34 measured FRET pairs (RMSD = 0.027, <ΔFRET> = –0.0025; Figure 5B and Supplementary Table S2, cf. the estimated experimental error, FRET_error_ = ± 0.025, see Materials and Methods). The fit to the experimental data was significantly worse for the best of the five static structures obtained from rigid-body docking (RMSD = 0.054), suggesting that the coarse-grained simulations better describe the experimental conformational ensemble and dynamics for these highly dynamic substrates.

The simulations of the free substrate identified two classes of structures: in ∼80% of configurations, both stacking interactions between the three nucleotides opposite the gap were maintained, resulting in a straighter geometry (Figure 5D, top). In ∼20% of configurations, at least one of these stacking interactions was broken, resulting in a state in which the system can explore a wide variety of bend angles (Figure 5D, bottom). FRET efficiencies were typically larger for unstacked configurations (Figure 5C – green circles), as the enhanced bending allowed the dyes to explore more proximal positions. Notably, although the stacked conformations are the dominant contributor to the FRET signal, excluding the unstacked states from the average FRET calculation lead to average computed FRET values significantly lower than those observed experimentally, worsening the agreement to the experiment (<ΔFRET> = –0.0025 with unstacked states versus –0.026 without unstacked states). This finding strongly suggests that bent states are present in the experimental ensemble for the gapped substrate. Bent states were also detected in all-atom MD simulations on the gapped substrate (Supplementary Figure S5H).

Encouraged by the excellent agreement between the computational and experimental results for the free substrate, we calculated free-energy landscapes from the relative abundance of conformations with specific bend angles (Figure 5E). The landscapes show that the angle seen in the Pol-bound state of the gapped substrate (∼120°) is accessible to the unbound gapped substrate (free energy difference of <4 kT); this bend angle is achievable only upon breaking at least one of the stacking interactions. However, once the stacking is broken, the substrate can freely explore a relatively flat landscape (Figure 5E, dashed lines), where the gap acts as a hinge. Simulations on nicked and duplex DNAs showed that it is harder for these substrates to adopt bend angles of 120° (Figure 5E), due to the increased energetic cost of breaking an additional stacking interaction and, in the case of the duplex, the extra chain connectivity constraints (>7 kT – for nicked DNAs, >>10 kT – for duplex DNAs).

We also inspected the coarse-grained simulations for evidence of melting of the downstream duplex DNA in the gapped substrate alone. In 28% of all configurations, we observed melting of the A-T base pair (fraying) at the downstream site immediately adjacent to the gap (Supplementary Figure S5F); when looking only at unstacked configurations, this fraction increased to 35%, partly due to the loss of a stabilizing cross-stacking interaction (Figure 5D). The second nucleotide was frayed in ∼5% of configurations irrespective of stacking state (Supplementary Figure S5F); fraying of three nucleotides was never observed. The propensity of fraying at the terminal base pairs of a duplex predicted by oxDNA is broadly consistent with the ranges suggested by previous experiments (44–46). In particular, as seen in Supplementary Figure S5F, the propensity to fray is relatively low for GC base pairs but is significantly enhanced for AT base pairs.

Taken together, our results are consistent with a conformational selection model in which Pol initially interacts with an unstacked, bent configuration of gapped DNA, a significant proportion of which is frayed by 1–2 nt around the gap.

### Bent DNA detected in live cells

**Figure 6.**
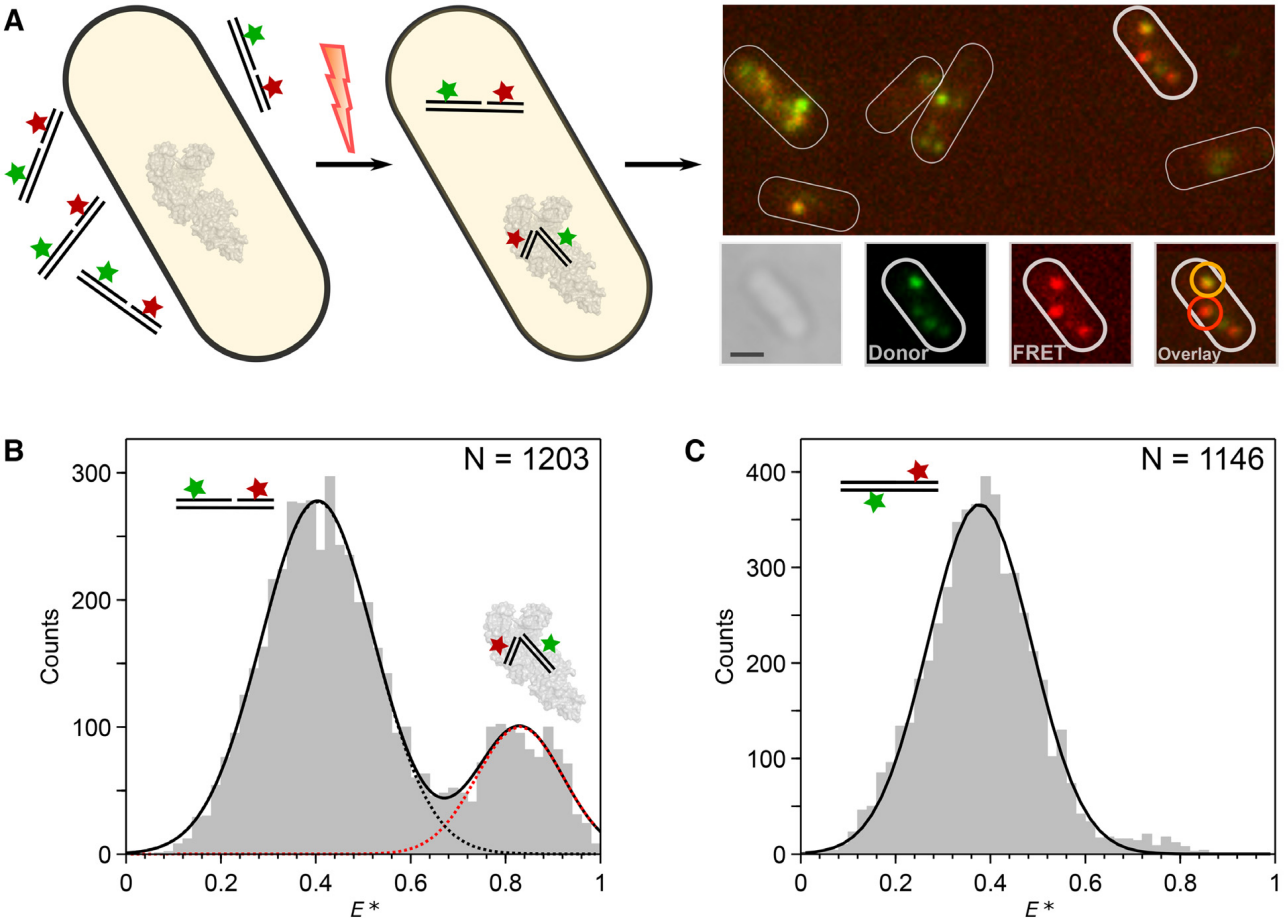
DNA bending detected in live cells. (**A**) Schematic showing internalization of doubly-labelled gapped DNA fragments into live *E. coli* using electroporation and single-molecule imaging (left to right). Example cell (bottom right) is shown in white-light image, donor fluorescence channel, FRET fluorescence channel, and latter both combined in overlay image. The overlay image is color-coded such that intermediate-FRET molecules appear orange and high-FRET molecules appear red; two example molecules are highlighted accordingly. Scale bar: 1 μm. (**B**) FRET histogram of tracked gapped DNA trajectories *in vivo*. Two major FRET species were observed for the T(–12)T(+8) substrate, which were attributed to unbent DNA (black dots; E* = 0.40) and bent DNA (red dots; E* = 0.83). The number of trajectories (*N*) is stated for each experiment. (**C**) FRET histogram of tracked duplex DNA trajectories *in vivo*. A single low-FRET species, was observed (black, E* = 0.38). See also Supplementary Figure S6.

To test for the existence of bent gapped DNA *in vivo*, as suggested by our binary structure, we measured the FRET efficiencies of individual labelled-DNA substrates in live *E. coli* cells. A small number of 1-nt gapped DNA molecules (1–5 molecules per cell) were internalized into cells by electroporation (47–49) and their FRET efficiencies monitored (Figure 6A; *Methods*).

We first internalized the T(–12)T(+8) gapped substrate, as it showed a large FRET change upon Pol binding *in vitro* (Supplementary Figure S6A and B). In live cells, we observed a bimodal FRET distribution consistent with the existence of both unbound (80%, *E* = 0.40) and bound (20%, *E* = 0.83) populations (Figure 6B). In contrast, a duplex control showed only a single, low-FRET peak (Figure 6C; cf. the *in vitro* data – Supplementary Figure S6C). The absence of a high-FRET population for this construct, which is not a substrate for the polymerase, is consistent with the interpretation that the high-FRET population observed with the gapped-DNA construct is a result of bending induced by the endogenous full-length Pol.

Whilst the labelling scheme above discriminated well between the FRET signals arising from unbound and bound DNA, we could not resolve the smaller FRET difference between the binary complex and high-FRET ternary complex seen in our *in vitro* work (Supplementary Figure S6B). We thus internalised the T(–18)T(+15) gapped substrate, which showed *in vitro* a larger FRET difference between the binary complex and the high-FRET ternary complex (Supplementary Figure S6D-E). The resulting FRET histogram from live cells lacked a significant high-FRET peak, but did exhibit two low-FRET peaks, consistent with the presence of unbound DNA, and DNA in the binary complex (Supplementary Figure S6F), and suggesting that little high-FRET ternary complex was present *in vivo*.

## DISCUSSION

The combination of single-molecule FRET with both coarse-grained and all-atom molecular simulations has provided substantial mechanistic and structural insight into the recognition and binding of DNA substrates by Pol. We have characterized the structure and dynamics of multiple species present in solution: the substrate alone, the binary complex and the high-FRET ternary complex. Further, we have obtained evidence for the *in vivo* relevance of the bent binary complex, detecting its FRET signature in live cells.

### Binary complex structure and dynamics

We obtained a unique, solution-based, high-precision structure (RMSD = 3.8 Å) of Pol bound to a gapped-DNA substrate, containing upstream and downstream duplex DNA flanking a 1-nt gap (Figures 2B and 3A). Previous structural efforts lacked any downstream duplex DNA and, as a result, its position and the conformation of the substrate were unknown. Our work showed that the gapped DNA in the binary complex structure adopted a 120° bend (discussed further below).

The location of the upstream DNA in the docked structure agrees very well with existing co-crystal structures containing primer-template substrates. This supports our rigid-body docking approach, and the accuracy of our positioning of the downstream DNA on the fingers subdomain. This positioning conclusively rejects early propositions that the DNA might be channelled through the cleft formed by the fingers and thumb subdomains (50,51). Our structure served as a starting point for all-atom MD simulations, which showed DNA dynamics in the binary complex, and identified transient DNA interactions with specific Pol residues. Some of these interactions involved residues implicated in previous biochemical studies, e.g. Y719 (32), providing a structural and mechanistic explanation for the experimental data; other residues (e.g. K549) revealed novel interactions that will merit further study.

### Y719 acts as a wedge in strand-displacement DNA synthesis

Our docked structure showed that the downstream DNA was positioned very close to Y719 (Figure 3D), confirming its involvement in strand displacement. DNA Pol I shares a three-helix bundle (O, O1 and O2) structural motif with T7 RNA polymerase (52). This motif participates in DNA binding and strand separation (53), and includes conserved residues Y719, S717 and R789 in *Bst* (F771, S769 and R841 in *E. coli*), which have been shown to be important for strand-displacement by Pol (32). This role for Y719 was further supported in our simulations, which showed the three-helix bundle (and particularly Y719) to be positioned between the template and non-template strands of the downstream DNA. The exact position of Y719 close to bases B(+1) and B(+2) on the downstream-template DNA is consistent with cross-linking data (54,55).

### Interactions with the downstream DNA

We also identified residues that interacted with the downstream DNA (R779 and R784; Figure 3E). These residues are highly conserved, with published sequence alignments showing 29 and 48 out of 50 bacterial polymerase sequences containing a homologous residue at positions 779 and 784, respectively (54). The two residues are likely to be functionally complementary, given their proximity in the structure and the similar interactions they form with downstream DNA in our simulations. Whereas our simulations indicate that R779 is more important for contacting DNA in the Bst Pol I, R784 may be the key residue in other bacterial polymerases that lack a positively charged residue at position 779, such as *E. coli* Pol. Interestingly, mutation of R784 to alanine (R836A in *E. coli*) has been shown to *increase* the binding of downstream DNA to the polymerase site (54,56), possibly due to R784 contributing to the bending and distortion of downstream DNA, or reflecting an unfavourable orientation of the side chain in the DNA–Pol binary complex.

K549 is part of a conserved motif (K)KT present in 33 out of 50 bacterial polymerase sequences analysed (54). In our simulations, interactions with K549 appear to keep the template strand away from its non-template counterpart, which may facilitate strand separation. Radioactive competition assays and cross-linking experiments have shown that Pol forms contacts with the first four nucleotides of the downstream template strand (54), which are beyond the reach of the active-site residues (Y714, S717, Y719 and R789), but could be accounted for by interactions with K549. The identity of the amino acid(s) cross-linking to base +4 could not be identified in these studies, likely due to the dynamics of the template strand and the transiency of interactions with K549, both features being apparent in our simulations.

### Downstream DNA is partially melted in the binary structure

The binary complex structure from rigid-body docking suggested that the downstream DNA cannot be fully base-paired proximal to the Pol fingers (Figure 2D). This idea was supported by our MD simulations, in which 4–5 nt of the downstream DNA remained single-stranded for the majority of the simulation time (Figure 3B). Our quenchable FRET assay confirmed that the downstream DNA is indeed melted when bound by Pol (Figure 4). When carrying out Okazaki fragment processing or long-patch base excision repair, Pol must perform strand-displacement DNA synthesis, replacing the RNA primer / damaged DNA with newly polymerized DNA. Our data suggest that the strand-displacement process starts before any DNA synthesis, with up to seven nucleotides being melted upon Pol binding to the substrate.

### Bent gapped-DNA detected *in vivo*

Our *in vivo* single-molecule experiments unequivocally show that non-extendable gapped-DNA constructs are bent in live *E. coli*, unlike duplex DNA. The close agreement between the FRET signatures of the bent species in cells and *in vitro* suggests that bending is likely mediated by the endogenous full-length DNA polymerase I binding, although the effect of other DNA-binding proteins cannot be excluded. For both internalized labelled DNAs, we observed a higher proportion of the lowest FRET species (corresponding to unbound DNA) than expected from our *in vitro* binding data and the expected cellular concentration of DNA Polymerase I (∼400 nM (57)). The high abundance of the low-FRET molecules in cells may reflect the effect of intracellular conditions (e.g. the presence of free nucleotides that can transiently occupy the 1-nt gap), the involvement of other proteins that could compete with the polymerase for gapped-DNA binding, or a lower affinity of the polymerase for gapped substrates *in vivo*.

Previous *in vitro* studies observed the presence of two molecules of Pol bound to DNA substrates (28–30). We also observed Pol_2_-DNA species in our *in vitro* titrations (Supplementary Figures S1 and S6), but not *in vivo*, suggesting that these complexes are unlikely to be important in the cellular context, where the presence of the 5′-nuclease domain in the full-length protein may inhibit dimer formation.

### Substrate structure and dynamics – a recognition signal?

Gapped DNA in the binary complex structure exhibited a 120° bend (Figures 2B and 3A). DNA bending was also observed in the crystal structure of the mammalian gap-filling DNA polymerase β, where the ∼90° bend observed was suggested to be important for the mechanisms of polymerisation and fidelity (6). Our data support the idea that bending may be a necessary mechanistic step for gap-filling polymerases, exposing more of the template base for interrogation by the incoming nucleotide. However, we propose bending may also play a role in substrate recognition and selectivity.

Our coarse-grained simulations on the free gapped DNA showed remarkable agreement with the smFRET data (Figure 5B) and have important implications for the binding mechanism of Pol. Since the breaking of the stacking interactions opposite the gap increases DNA bendability, unstacking will likely occur as a step on the path to Pol binding. In addition, the high flexibility of the unstacked DNA suggests that the substrate can adopt a close-to-final bent conformation even prior to Pol complex formation. The simulations also provide an explanation for Pol substrate specificity, specifically its increasing binding preference for gapped over nicked DNA, previously observed by gel shift assays (28) and ensemble anisotropy (58). This preference appears to arise from the increased flexibility of the gap over the nicked DNA, reflected in the different energy cost required for their bending. In this way, the substrate specificity is encoded in the structure and dynamics of the DNA substrate itself, allowing sequence-unspecific recognition of gapped DNA by Pol.

Interestingly, other forms of DNA modification can affect DNA flexibility; cytosine methylation reduces flexibility, while 5-formylcytosine (a substrate for base excision repair) was shown to increase flexibility (59). Thus, it is likely that increased DNA flexibility may act as a general recognition signal for a variety of DNA repair processes.

### Gapped DNA recognition: conformational capture followed by an ‘on-protein’ rearrangement

**Figure 7.**
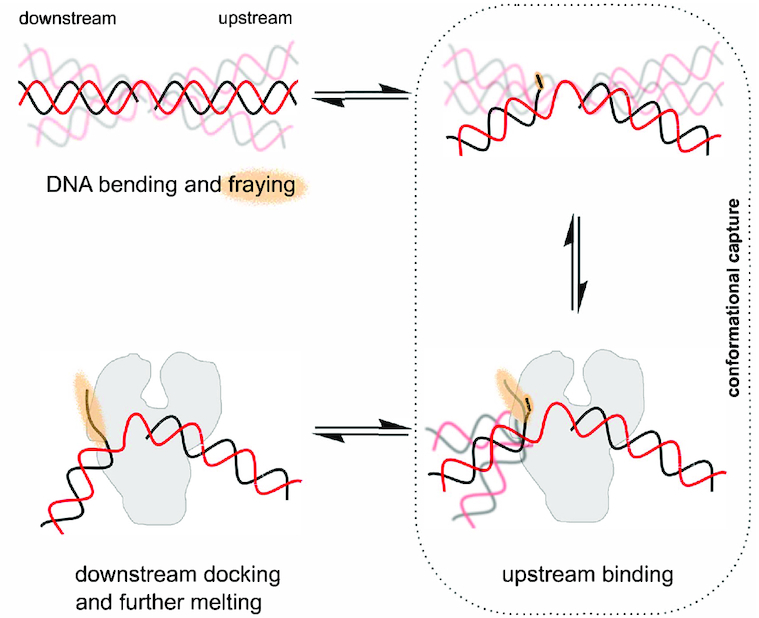
Gapped DNA recognition: conformational capture followed by an ‘on-protein’ rearrangement. Gapped DNA is dynamic adopting bent and frayed states (orange haze). Pol can bind to the upstream DNA when the downstream DNA conformation is not impeding the Pol (conformational capture of slightly bent states). Following binding of the upstream DNA, the downstream DNA now docks and is further melted, beginning the process of strand-displacement.

Based on our results, we propose the following model for recognition and binding of a gapped DNA substrate by Pol involving conformational capture followed by an ‘on-protein’ rearrangement (Figure 7). The DNA substrate rapidly interconverts between stacked and unstacked states; the unstacked conformations are generally more bent and show increased fraying 1–2 nt around the gap. The degree to which fraying of the downstream duplex is important in the recognition process, is likely to be sequence dependent. Pol initially interacts with the upstream DNA while the substrate is in an unstacked state (conformational capture). This upstream region of the substrate resembles a primer-template structure, which is known to bind tightly to Pol (*K*_D_ < 1 nM; (54) forming a sufficiently stable complex for crystallization (16,31). This conformational selection step does not necessarily require the substrate to adopt the precise 120° bend angle seen in the binary complex; rather, the DNA conformational flexibility helps to avoid blocking binding through steric clashes. Having bound the upstream duplex, the downstream duplex is free to sample conformational space (as seen in the MD simulations on the binary complex; Figure 3A), docking to the protein, and fraying the additional 3–4 nts, resulting in the complete binding of the gapped DNA (*K*_D_ = 0.4 nM; Supplementary Figure S1A). This proposed two-step binding mechanism comprises an initial conformational selection step in which the substrate is bound, followed by an ‘on-protein’ conformational search, in which the DNA and the protein both search conformational space.

Our results cannot rule out alternative models where Pol I binds to a non-bent 1-nt-gap DNA conformation, and then waits for fraying and/or bending to occur (an on-protein conformational search, like the second state of the model in Figure 7). However, the structure we obtained for the binary complex, and the low affinity of Pol I for non-bent DNA substrates (using Pol binding to linear dsDNA as a proxy for the 1-nt gap linear conformation) suggest that initial binding to transient bent states is more likely.

Other structure-specific DNA binding proteins which have been shown to interact with bent DNA (e.g. FEN1, Pol β) are also likely to exploit the conformational dynamics of their substrates for recognition and binding, as was also recently suggested for DNA mismatch recognition (60). Thus, the mechanism we propose (initial conformational selection step, sensing the increased flexibility of the substrate DNA, followed by an ‘on-protein’ rearrangement), may be generally applicable to many structure-specific DNA binding enzymes, especially for DNA repair enzymes, which search vast regions of undamaged DNA rapidly to identify and fix sites of DNA damage to maintain genomic integrity and normal cellular function.

## Supplementary Material

gkz797_Supplemental_Files

## Notes

Present address: Timothy D. Craggs, Sheffield Institute for Nucleic Acids, Department of Chemistry, University of Sheffield, S3 7HF, UK.

Present address: Majid Mosayebi, School of Mathematics, University of Bristol, University Walk, Bristol BS8 1TW, UK.

Present address: Anne Plochowietz, Palo Alto Research Center, 3333 Coyote Hill Rd, Palo Alto, CA 94304, USA.
