# Supplementary material for: Substrate conformational dynamics facilitate structure-specific recognition of gapped DNA by DNA polymerase
Source: Nucleic Acids Res. 2019 Sep 23;47(20):10788–800. doi: 10.1093/nar/gkz797 (PMC6846080; doi:10.1093/nar/gkz797)
Supplement: gkz797_Supplemental_Files [file gkz797_supplemental_files.zip › supplemental information revised.pdf]

Substrate conformational dynamics facilitate structure-specific recognition of gapped DNA by DNA polymerase

Craggs et al.

SUPPLEMENTAL INFORMATION

Supplemental Materials and Methods

Figures:

S1 DNA-Pol equilibrium model and example 'ES' histograms

S2 Rigid-body docking and high-FRET Pol_2-DNA ternary complex model

S3 Molecular dynamics

S4 Quenchable FRET

S5 Free DNA substrate structure and dynamics

S6 *In vivo* detection of bent gapped DNA

Tables:

S1 Binary complex and high-FRET ternary complex FRET distances

S2 Free DNA substrate FRET distances: measured and modelled

S3 Oligonucleotide sequences for assembling the 1-nt gapped substrates

S4 R_0 determination and measured anisotropy values

Movies:

M1 Binary complex all-atom MD trajectory.mov

M2 1-nt gapped DNA OxDNA simulation.wmv

PDB file:

P1 Pol-DNA_Binary-Structure.pdb

A representative snapshot from the all-atom MD simulations providing the coordinates of all protein and DNA atoms in pdb format.

SUPLEMENTARY MATERIALS AND METHODS

Protein Expression, purification and labelling. Pol variants were expressed from an N-terminal-His6, D424A construct and purified as described (1). The D424A mutation inhibits the proof-reading exonuclease activity. Briefly, a plasmid carrying the gene encoding Pol was transformed into HMS174 (DE3) cells, and single colonies inoculated in 25 ml LB, supplemented with 50 µg/ml carbenicillin. The cultures were grown over-night at 220 rpm and 37°C, and were used to inoculate 1 liter of LB supplemented with carbenicillin. The culture was grown to an OD600 of 0.6, at which point expression was induced with 0.5 mM Isopropyl β-D-1-thiogalactopyranoside (IPTG). After 2 hours of expression, the cells were harvested by centrifugation (20 min at 3000 rpm and 4 °C; GS-6R Beckman), resuspended in cold 50 mM Tris pH 7.5, and spun down in an ultracentrifuge (15 min at 8,000 rpm at 4 °C; Sigma 3K30, rotor 12150-H). Finally, the pellet was resuspended in lysis buffer (50 mM Tris pH 7.2, 300 mM NaCl, 1 mM β-mercaptoethanol, 10 mM imidazole, 2 mg/ml lysozyme and 0.02 mM Phenylmethane sulfonyl fluoride; PMSF). The cells were stored in the lysis buffer overnight at -80°C.

The frozen cells were thawed and fresh PMSF (25 µl) was added. Cells were lysed by sonication (6 cycles of 5-sec ON and 10-sec OFF time) and the cell debris was spun down (20 min at 15,000 rpm at 4 °C; Sigma 3K30). The supernatant containing the cell lysate was combined with Ni-NTA resin (pre-equilibrated in buffer A: 50 mM Tris pH 7.2, 300 mM NaCl, 1 mM β-mercaptoethanol and 10 mM imidazole), and the protein allowed to batch-bind (1 hour at 4°C). The resin was spun down, resuspended in buffer A, applied to a plastic column and washed with buffer A containing increasing concentrations of imidazole (10, 20, and 27 mM). The protein was eluted in buffer A containing 100 mM imidazole, and the fractions analyzed by absorbance and SDS-PAGE. The concentrated fractions were pooled, and dialyzed into 50 mM Tris pH 7.2, 1 mM dithiothreitol (DTT) overnight at 4 °C. The dialyzed samples were combined in a 1:1 ratio with 2x glycerol storage buffer (80 % glycerol, 50 mM Tris pH 7.2, 2 mM DTT) and stored at -20°C.

Pol variants containing a single cysteine (C907+, C907S / K550C and C907S / L744C) were labelled using a maleimide derivative of Cy3B (GE Healthcare) as described (1). Briefly, purified Pol samples were reduced (5 mM DTT, 1 hr at 22°C), and DTT was removed by dialysis into 50 mM Tris pH 7.1, 0.12 mM tris(2-carboxyethyl)phosphine (TCEP). A two-fold excess of the Cy3B maleimide dissolved in DMSO was added to the

protein sample and the reaction allowed to proceed overnight at 4 °C with gentle rocking. The reaction was quenched with 1 mM DTT, and applied to a heparin column (pre-equilibrated in heparin buffer containing 20 mM Tris pH 7.4, 1 mM ethylenediaminetetraacetic acid (EDTA), 2 % glycerol and 1 mM β-mercaptoethanol), washed with heparin buffer containing 50 mM NaCl, and the protein eluted in buffer containing 400 mM NaCl. Samples were dialyzed first into 1 liter of 50 mM Tris pH 7.4, 25 mM NaCl, 1 mM DTT, for 3x 1 hour, and then into 500 ml of the same buffer containing 40 % glycerol, overnight, before storing at -20 °C. Labelling efficiencies (typically ~80 %) were determined by UV-Vis absorbance, using extinction coefficients for Pol (58,790 M^{-1} cm^{-1} at 280 nm) and Cy3B (130,000 M^{-1} cm^{-1} at 570 nm), and taking into account the Cy3B absorbance at 280 nm.

DNA labelling and annealing. DNA oligonucleotides (oligos; Table S3) were prepared using automated synthesis (IBA GmbH), and labelled with NHS-ester derivatives of Cy3B (GE Healthcare) and Atto647N (Atto-tec) via dT-C6-amino linkers at selected positions according to the manufacturers' protocols. Labelled oligos were purified by 20% polyacrylamide gel electrophoresis. Bands were visualized by UV-shadowing, cut, and extracted from the gel using an overnight crush and soak protocol at 4˚C. The sample volume was reduced (by centrifugal evaporation) and buffer exchanged into TE buffer (Microbiospin6 columns, BioRad). Gapped-DNA substrates were assembled by annealing three single-stranded oligos (one from each group - DNA1, DNA2 and DNA3; Table S3) in annealing buffer, 20 mM Tris-HCl pH 8.0, 100 mM NaCl, and 1 mM EDTA. Samples were heated to 94°C and subsequently cooled to 4°C, in steps of 10°C over 45 min. Annealed substrates were stored at -20°C. For DNAs prepared for electroporation, the oligonucleotides were annealed in a low-salt annealing buffer (20 mM Tris-HCl (pH 8.0), 10 mM NaCl, 1 mM EDTA).

Single-molecule FRET measurements. Single-molecule FRET measurements were performed at room temperature using a home-built confocal microscope with 20 kHz alternating-laser excitation between a 532-nm (Samba, Cobolt, operated at 240 µW) and a 638-nm laser (Cube, Coherent, operated at 60 µW), coupled to a 60x, 1.35 numerical aperture (NA), UPLSAPO 60XO objective (Olympus). For DNA-DNA

measurements, labelled DNA was present at < 100 pM and unlabelled Pol (when present) at 3 nM concentration. For Pol-DNA measurements, both Pol and DNA were present at 100 pM concentration. Measurements were taken in 'Pol buffer', consisting of 40 mM 4-(2-hydroxyethyl)-1-piperazineethanesulfonic acid (HEPES)-NaOH, pH 7.3, 10 mM $MgCl_2$, 1 mM DTT, 100 μg ml^{-1} bovine serum albumin, 5% (vol/vol) glycerol, 1 mM mercaptoethylamine. We recorded 3-6 datasets of 10 min for each distance measurement and combined for analysis. Photon streams in DD, DA and AA channels were recorded and processed using custom-written software (LabVIEW). Bursts were filtered for the correct labelling stoichiometry **(2)**, and accurate FRET was calculated as described below. FRET histograms were fitted to single, double or triple Gaussian functions.

Derivation of the multistate equilibrium model. A three-species model, in which the observed low-, mid-, and high-FRET states correspond to DNA alone, DNA-Pol binary complex, and a dimer DNA-Pol_2 respectively, could not account for the persistence of the mid-FRET signal at high Pol concentrations (Fig S1A). The simplest model that could account for all the data involved a second dimer species with a FRET efficiency indistinguishable from the DNA-Pol binary complex (Fig S1B). In this model, binding of Pol to the DNA, forming the binary complex (Pol:DNA*), is described by the association constant , K_1=[Pol:DNA*]/[DNA]. Binding of a second Pol to the binary complex yields a mid-FRET dimer species (Pol_2:DNA*) governed by the association constant K_2=[Pol_2:DNA*]/[Pol:DNA*]. This mid-FRET dimer can isomerize to the high-FRET dimer (Pol_2:DNA**), a process described by the equilibrium constant K_3=[Pol_2:DNA**]/[Pol_2:DNA*]. The corresponding dissociation constants are defined as $K_{D1}=1/K_1$ for the formation of the binary complex and $K_{D2}=1/(K_1K_2)$ for the formation of the mid-FRET dimer species.

The total DNA substrate concentration is:

$$\begin{aligned}[\text{Total}] &= [\text{DNA}] + [\text{Pol:DNA*}] + [\text{Pol}_2\text{:DNA*}] + [\text{Pol}_2\text{:DNA**}] \\ &= [\text{DNA}] + K_1[\text{Pol}][\text{DNA}] + K_1K_2[\text{Pol}]^2[\text{DNA}] + K_1K_2K_3[\text{Pol}]^2[\text{DNA}] \qquad \textbf{(S1)}\end{aligned}$$

The fractions of each species as a function of Pol concentration are:

$$[DNA]/[Total] = 1/(1 + K_1[Pol] + K_1K_2[Pol]^2 + K_1K_2K_3[Pol]^2) \quad \textbf{(S2)}$$

$$[Pol{:}DNA^*]/[Total] = K_1[Pol] /(1 + K_1[Pol] + K_1K_2[Pol]^2 + K_1K_2K_3[Pol]^2) \quad \textbf{(S3)}$$

$$[Pol_2{:}DNA^*] /[Total] = K_1K_2[Pol]^2/(1 + K_1[Pol] + K_1K_2[Pol]^2 + K_1K_2K_3[Pol]^2) \quad \textbf{(S4)}$$

$$[Pol_2{:}DNA^{**}] /[Total] = K_1K_2K_3[Pol]^2/(1 + K_1[Pol] + K_1K_2[Pol]^2 + K_1K_2K_3[Pol]^2) \quad \textbf{(S5)}$$

The fractional populations of the low- mid and high-FRET states are:

$$\text{Low-FRET} = [DNA]/[Total] = 1/(1 + K_1[Pol] + K_1K_2[Pol]^2 + K_1K_2K_3[Pol]^2) \quad \textbf{(S6)}$$

$$\text{Mid-FRET} = [Pol{:}DNA^*]/[Total] + [Pol_2{:}DNA^*] /[Total]$$
$$= K_1[Pol]\ (1 + K_2[Pol])/(1 + K_1[Pol] + K_1K_2[Pol]^2 + K_1K_2K_3[Pol]^2) \quad \textbf{(S7)}$$

$$\text{High-FRET} = [Pol_2{:}DNA^{**}] /[Total]$$
$$= K_1K_2K_3[Pol]^2/(1 + K_1[Pol] + K_1K_2[Pol]^2 + K_1K_2K_3[Pol]^2) \quad \textbf{(S8)}$$

We used a global fitting approach to fit the variation in the fractional populations of the three FRET states simultaneously as a function of Pol concentration, and to determine the equilibrium constants K_1, K_2, and K_3. From the equilibrium constants and their standard errors, we calculated the dissociation constants.

Accurate FRET corrections. The apparent FRET efficiency, E* was calculated from the DA and DD photon streams:

$$E^* = DA / (DD + DA) \quad \textbf{(S9)}$$

Similarly, the apparent stoichiometry, S* was calculated using:

$$S^* = (DD +DA) / (DD + DA + AA) \quad \textbf{(S10)}$$

To obtain the accurate FRET efficiency, the raw photon streams were sequentially corrected for background counts, cross talk, and gamma / beta factors (which take into

account the different detection efficiencies, quantum yields and excitation cross sections of the two dyes), as described (3, 4).

First, the three photon streams were corrected for background, which arises from impurities, Raman scattering from the solvent and dark counts in the detectors. For each burst, the corrected counts were calculated from the raw counts by subtracting the background count rate, multiplied by the length of the burst. Typical background count rates were 1-3 photons per ms.

After the background correction, the leakage fraction of the donor emission into the acceptor detection channel and the direct excitation of the acceptor by the donor-excitation laser were obtained. The correction factor for leakage (lk) was determined from the FRET efficiency of the donor-only population, $E_{don\text{-}only}$:

$$lk = 1/(1/ E_{don\text{-}only} - 1) \qquad \textbf{(S11)}$$

The correction factor for direct excitation (dir) was determined from the apparent stoichiometry value of the acceptor-only population, $S_{acc\text{-}only}$:

$$dir = 1/(1/S_{acc\text{-}only} - 1) \qquad \textbf{(S12)}$$

The DA intensities and the FRET efficiency and stoichiometry were then corrected as follows:

$$DA_{corr} = DA - DD*lk - AA*dir \qquad \textbf{(S13)}$$

$$E_{PR} = (DA_{corr}) / (DA_{corr} + DD) \qquad \textbf{(S14)}$$

$$S_{PR} = (DA_{corr} + DD) / (DA_{corr} + DD + AA) \qquad \textbf{(S15)}$$

Finally, the gamma and beta parameters were obtained from a linear fit to a plot of $1/S_{PR}$ vs E_{PR}:

$$1/S_{PR} = slope*E_{PR} + intercept \qquad \textbf{(S16)}$$

$$\beta = intercept + slope – 1 \qquad \textbf{(S17)}$$

$$\gamma = (intercept - 1)/(intercept + slope -1) \qquad \textbf{(S18)}$$

The fully-corrected accurate FRET efficiencies and stoichiometries, E and S are given by:

$$E = (DA_{corr}) / (DA_{corr} + \gamma^*DD) \quad \textbf{(S19)}$$

$$S = (DA_{corr} + \gamma^*DD) / (DA_{corr} + \gamma^*DD + AA/\beta) \quad \textbf{(S20)}$$

Accurate determination and application of all correction parameters was checked visually on the ES histograms, as all FRET populations should be located at S~0.5. Gamma and beta factors were determined separately for DNA-DNA and DNA-Pol measurements. This was necessary because of the significant difference in the quantum yield of the donor when attached to DNA or protein (see below and Table S4).

Conversion of accurate FRET to distance. Accurate FRET efficiency E, was converted to distance R, according to the equation:

$$E = 1 / (1 + [R/R_0]^6) \quad \textbf{(S21)}$$

using experimentally determined values for the Förster radius, R_0, which were calculated according to the equation:

$$R_0^6 = \frac{9000 \ln(10) Q_D \kappa^2 J}{128\, \pi^5 N_A n^4} \quad \textbf{(S22)}$$

where Q_D is the quantum yield of the donor (which must be measured; see below), N_A is Avogadro's number, and n is the refractive index of the medium. The term κ^2 describes the relative orientation of the transition dipoles of the donor and acceptor. Its value lies in the range of 0-4, and it is often assumed to be equal to 2/3, which is the case when both fluorophores have unrestricted rotational freedom (5).The overlap integral J is a measure of the degree of overlap between the donor emission and acceptor excitation spectra (6), and can be calculated according to:

$$J(\lambda) = \int_0^\infty F_D(\lambda)\varepsilon_A(\lambda)\lambda^4 d\lambda \quad \textbf{(S23)}$$

where F_D is the corrected donor fluorescence intensity at a particular wavelength λ, with the total intensity normalized to unity, and ε_A is the extinction coefficient of the acceptor at the same wavelength.

Quantum yields were measured according to established methods (7, 8) for the following donor samples: free Cy3b-maleimide dye, Cy3b attached to gapped DNA (in the presence and absence of unlabelled Pol in a 1:1 molar ratio), and Cy3b attached to different positions of Pol (K550, L744, C907). Each sample was diluted from a glycerol stock to 5 µM final concentration in Pol buffer. Free Cy3b-maleimide dye was reduced with 10 mM DTT for 10 min prior to dilution. Absorbance at 490 nm was recorded for each sample, using a UV-visible spectrophotometer (Cary 50 Bio, Varian). An emission scan was taken of the same sample using a steady-state fluorimeter (PTI), exciting at 490 nm and recording at 510-700 nm. Samples were diluted and recordings repeated 5 times, to populate absorbance in the 0 to 0.1 region, where absorbance and emission are linearly related. The same procedure was applied to the reference dye, rhodamine 6G, dissolved in ethanol. The quantum yield of the donor dye was then calculated according to equation:

$$Q_D = \frac{Q_R E_D A_R n_D^2}{A_D E_R n_R^2} \qquad \textbf{(S24)}$$

where *Q* is quantum yield, *E* is integrated emission across the whole spectrum, *A* is absorption at 490 nm, *n* is the refractive index of the medium, and *D* and *R* refer to the donor and the reference dyes, respectively. Established values were taken for the quantum yield of rhodamine 6G in ethanol (0.95; (9) and for the refractive index of ethanol (1.361). To calculate the overlap integrals, absorption spectra of the following acceptor samples were also measured: Atto647 free dye, Atto647N attached to DNA (in the presence and absence of Pol in a 1:1 ratio), and Atto647N attached to Pol. Samples were diluted in Pol buffer to 2 µM concentration, and absorption recorded at 400-710 nm. Both the absorption spectra of the acceptor, and the emission spectra of the donor (see above) were corrected for background, normalized, and the overlap integral calculated as in equation 3.3. The extinction coefficient of Atto647N at A_{max} was taken as 150,000 $M^{-1}cm^2$ (from manufacturer's website; http://www.atto-tec.com). This allowed isotropic R_0 values to be calculated (Equation S22), assuming orientational averaging (κ^2 = 2/3) and the refractive index of water (n=1.333).

To test if orientational averaging is justified, steady-state anisotropies were measured (7). Samples were diluted to 100 nM in Pol buffer and excited with vertically polarized light at 532 nm (donor) or 638 nm (acceptor samples) in a steady-state fluorimeter (PTI). Fluorescence was measured through horizontally and perpendicularly oriented emission filters at 570 nm (donor) or 669 nm (acceptor samples) over 1 minute. Anisotropy values (Table S4) were calculated from the difference of vertically and horizontally polarized emission intensities, corrected for background and for the different sensitivities of the emission channel for vertically and horizontally polarized light.

Distance calculations and accessible volume modelling of dye positions. Accurate FRET efficiencies were converted to their corresponding distances using a FRET error of +/- 0.025 (determined from the standard error of the mean in three independent FRET measurements of the same sample), and using experimentally determined R_0 values (Table S4). The R_0 values used were 64.5 Å for DNA-DNA and for 59.0 Å for DNA-Pol distances, and the error in R_0 was assumed to be the error that was propagated from the uncertainty in quantum yield determination of +/- 0.10. The experimentally determined distances correspond to FRET-averaged distances, $\langle R_{DA} \rangle_E$ in the accessible volume model established by the Siedel laboratory (10). In this model, dye rotation occurs faster than the FRET process, but the position of the dye is fixed on the timescale of FRET. Other dye modelling methods based on Bayesian statistics can also be used (Muschielok et al., 2008).

The FRET-averaged distance is calculated by averaging the distances between individual dye positions in the donor and acceptor accessible volumes. For rigid body docking, these FRET-averaged distances were converted to distances between mean dye positions R_{mp}, using a third-order polynomial function:

$$R_{mp} = 26.23305+1.85509*\langle R_{DA} \rangle_E -0.00938*\langle R_{DA} \rangle_E{}^2+0.000035569*\langle R_{DA} \rangle_E{}^3 \qquad \textbf{(S25)}$$

that was established by calculating R_{mp} and $\langle R_{DA} \rangle_E$ values for pairs of dyes at different positions along a double-stranded DNA using, as described (11). The radii, linker lengths and linker widths of Cy3B and Atto647N dyes were estimated from their structures in silico using ChemDraw (Perkin Elmer) and are given here (in Å):

Dye	Linker length	Linker width	Radius 1	Radius 2	Radius 3
Cy3B (DNA)	14.2	4.5	8.2	3.3	2.2
Cy3B (Protein)	9.1	4.5	7.7	2.5	1.3
Atto647N (DNA)	17.8	4.5	7.4	4.8	2.6

We used the accessible volume (AV) algorithm of the FPS software (11) to model the mean positions of the dyes for each Pol and DNA attachment site. The attachment points were taken to be the S atoms of Cys residues, and the C7 atoms of dTTP residues. For quFRET (see below) we calculated the percentage of the donor accessible volume that overlapped with the acceptor accessible volume using custom written code in MATLAB (Mathworks). Accessible volume elements were counted as overlapping if the distance between them was smaller than the lattice spacing used to calculate the initial AV.

Rigid body docking. The polymerase structure was obtained from the *Bst* X-ray crystal structure (PDB code 1L3U; Johnson *et al.*, 2003). DNA was removed and Cys substitutions were introduced at positions K498, V692 and A855 (corresponding to *E. coli* residues K550, L744 and C907) using the PyMOL Molecular Graphics System, Version 1.8 Schrödinger, LLC. B-DNA models of the upstream and downstream DNA were made using 3D-DART modelling server (12) and were truncated at the gap-proximal ends by 3 base-pairs each for the purposes of rigid-body docking. Three-body rigid-body docking with Pol, upstream and downstream DNA structures was performed in the FPS software (11) using the calculated R_{mp} distances. Docking was repeated 1000 times from different starting configurations of the binding partners, using a clash tolerance of 6 Å. This treatment generated several clusters of structures, which were distinguished by the different RMSD values relative to each other, and the different goodness of fit to experimental data assessed by the reduced chi-squared parameter (χ_r^2). Structures with χ_r^2 values above 6 were rejected, and one structure from each of the remaining clusters was further refined using a clash tolerance of 2 Å, and then again using a tolerance of 1 Å, during which steps the AV clouds were recalculated. The structure with the lowest χ_r^2 was taken, and the R_{mp} distances from the model back-converted to $\langle R_{DA} \rangle_E$ and FRET efficiency values, to compare with the experimental FRET data. For precision estimation,

100 bootstrapped structures were generated from the best model, using a clash tolerance of 1 Å. The coordinates of each P atom were extracted, and the RMSD of each P atom calculated across the 100 bootstrapped structures.

To compare the position of the upstream DNA in the docked structure with the crystal structure, the protein components of the FRET-restrained and crystal structures were aligned in PyMol. The RMSD of the upstream DNA fragment between the two structures was calculated as for bootstrapped structures, but across all P atoms. The DNA structure in complex with the Pol dimer was obtained using the same procedure as the Pol-DNA structure, but with no polymerase present in the docking. In the case of the DNA structure in the absence of Pol, the DNA fragments were at their full length, and with an additional distance restraint of 5 +/- 2.5 Å imposed between the C atoms in the template strand opposite the gap, to account for the covalent link between the two.

All-atom molecular dynamics simulation - Model preparation. The protein atoms and the catalytic magnesium ion were extracted from the *Bst* X-ray structure PDB file (code 4BDP; (13)). The online server 'WHAT IF' was used to check for errors in the PDB, and build the missing side chains into the structure (14, 15). PyMol was used to align the FRET-restrained structure with the X-ray structure, based on the protein component only. The downstream DNA in the docked structure was extended to its full length, and its template strand linked to the template strand of a 5-nucleotide fragment of upstream DNA from the X-ray structure (which also includes the templating nucleotide and one nucleotide downstream). Refinement of the docking pose was performed such that the conformation of the DNA backbone was not significantly disturbed, and no steric clashes occurred with the polymerase, which resulted in 6 base-pairs of downstream DNA being unpaired. The upstream DNA fragment was then extended using the sequence of upstream DNA from the docked structure. This step was justified by the excellent agreement in the position of the upstream DNA between the X-ray and docked structures (Fig S2B). For DNA-only simulations, DNA models were generated using the 3D-DART server (12). DNA atoms were extracted from the PDBs and terminal phosphate groups removed using PyMol. In the case of gapped DNA, the central nucleotide was removed and a 5' phosphate group generated instead.

Force fields and parameters. All complex simulations and high-temperature DNA simulations were run using Amber ff99sb force field (16) with modified nucleic acid

parameters (parmbsc0; (17, 18). The crystal structure control and the DNA-only simulations, were run using Amber ff99sb-ILDN with Amber94 nucleic acid parameters (19). No parameters were available in either force field for the 5' phosphate groups of DNA, as these are usually missing in crystal structures due to their high flexibility. The phosphate group had to be modelled at the 5' end of the gap in our DNA substrate, as it is both physiologically relevant and present in our single-molecule experiments. Therefore, the force fields were modified by assuming that the parameters of the β-phosphate of free ADP available online (http://research.bmh.manchester.ac.uk/bryce/amber/), are a reasonable approximation for the α-phosphate of the gap-proximal dDTP.

Simulation conditions. All simulations were carried out using Gromacs 4.6 (20). The X-ray structure control simulations and complex simulations were done using explicit solvent (TIP3P) in a triclinic box, with a minimum 10-Å solvent edge, in the presence of 10 mM $MgCl_2$. The system was neutralized with addition of magnesium ions, and energy-minimized using steepest descent minimization. In order to stabilize the temperature of the system, equilibration was performed in the NVT ensemble for 100 ps, with the temperature of 298 K maintained using a Berendsen thermostat (21). Next, the pressure of the system was stabilized by equilibration in the NPT ensemble for 1 ns, with the temperature of 298 K and the pressure of 1 bar retained using a V-rescale thermostat (22) and a Berendsen barostat (21), respectively. During equilibration, DNA, protein heavy atoms and the catalytic magnesium ion were position-restrained with a force constant of 1,000 $kJmol^{-1}nm^{-2}$. DNA was equilibrated for an additional 10 ns with protein heavy atoms restrained, under the NPT conditions. Atom velocities were preserved between the equilibration steps, and between equilibration and production steps. Unrestrained production was finally allowed to run for 100 ns, with the temperature of 298 K and the pressure of 1 bar maintained by the V-rescale thermostat and a Parrinello-Rahman barostat (23). Periodic boundary conditions and the Verlet cut-off scheme were used, and long-range electrostatic interactions were accounted for by the Particle-Mesh Ewald method (24). All bonds were treated as constraints with the LINCS algorithm, resulting in a time step of 2 fs. Coordinates were saved to an output trajectory every 5 ps. Repeat simulations were carried out using different randomly numbered seeds, generating different initial atom velocities each time.

In the case of full-length DNA-only simulations, the conditions were the same except that a square box was used, with dimensions equal to the length of the DNA plus a 10-Å solvent

edge. The NVT and NPT equilibration steps were performed, and the production times were 20 ns. In the case of high-temperature DNA simulations carried out as part of model preparation, the conditions were the same as for the complex simulations except that the temperature during the equilibration and production runs was 400 K, and the production times were 2 ns. All DNA heavy atoms were position-restrained during the production runs, except for the 6 base pairs in the protein-proximal, downstream part of the DNA, which were unpaired in the starting configuration.

Analysis. All analysis was carried out using Gromacs 4.6 or 5.0, and VMD (25). Trajectories were repaired for periodic boundary conditions, and processed to include only every 10th frame, corresponding to 50-ps steps. Maps of occupancy of DNA and of polymerase residues during the simulation were created with VMD's volmap density function, using an isovalue of 0.001. RMSD and end-to-end distance measurements were done using standard functions in Gromacs. The flap-to-template H-bonds were quantified by measuring the number of bonds at any one time in the simulation, using a distance cut-off of 0.33 nm, and an angle cut-off of 30°. The position of residue Y719 relative to the DNA was calculated by measuring the distance between the centers of mass of Y719 side chain and individual DNA residue base moieties. Pol-DNA interactions were detected by measuring the minimum distance between any nitrogen atom of a specific Pol residue and a specific phosphorous atom in DNA, during the entire simulation. Distances below 0.4 nm were taken as indicating an interaction.

Coarse-grained molecular dynamics simulation of DNA substrates using oxDNA. DNA substrate systems were simulated using oxDNA, a nucleotide-level coarse-grained model of DNA in which each nucleotide is modelled as a rigid body. The oxDNA model has been described in detail (26, 27) and is implemented in a simulation package which is available for download (http://dna.physics.ox.ac.uk/). It was designed to reproduce the thermodynamic and mechanical properties of both single- and double-stranded DNA (26, 28), and has proven powerful in predicting the kinetics of the basic dynamical processes in DNA systems (29–31). Therefore, it is particularly suited for probing the structure and dynamics of the DNA substrates in this study. The oxDNA interaction potential consists of terms representing the backbone connectivity (modelled as a finitely-extensible nonlinear elastic spring), excluded volume, hydrogen bonding between Watson-Crick (WC)

complementary base pairs, stacking between adjacent bases along the chain, coaxial stacking between non-adjacent bases, and cross stacking (Fig S5E). Aside from backbone connectivity and excluded volume, all interactions are anisotropic, depending on the relative orientation of the nucleotides. Orientational modulations of the stacking potential favors the bases to form coplanar stacks, and hydrogen bonding can occur between complementary WC base pairs when they are anti-aligned, leading to the formation of double-helical structures for which the helical twist arises from the different length scales of the backbone separation and the optimal stacking separation. Within oxDNA, the bases in the single-stranded DNA can stack/unstack and the strengths of hydrogen bonding and stacking interactions depend on the identities of the interacting bases (27). The model has been parameterized for a Na^+ concentration of 0.5 M. Based on commonly used scaling relationships, the hybridization thermodynamics under these conditions are expected to be very similar to the experimental buffer conditions in this study. For example, using the relationship in Ref. (32) the experimental conditions are expected to map onto an equivalent system with $[Na^+]$=0.42 M. Furthermore, at these relatively high salt conditions, the thermodynamic properties vary slowly with $[Na^+]$.

We performed 100 simulations of 10^8 steps each for each of the gapped, nicked and duplex DNAs, with interaction energies and configurations sampled every 10^3 steps. The time step was 0.005 simulation units, where one simulation unit implies a time of 3.03 x 10^{-12} s. The temperature was set to 295 K, and an Andersen-like thermostat was used (33). Particle velocities were refreshed every 10^3 steps from the Maxwell distribution corresponding to the simulation temperature, with fixed probabilities of 0.02 and 0.0067 for the linear and angular velocities, respectively.

The bend angle was calculated from the vectors placed along the midlines of the two helical segments, as described previously (34). The relative free energies were calculated from the MD trajectories, as follows:

$$A(|\theta|)/k_B T = -\log\left(\frac{p(|\theta|)}{p(|\theta_0|)}\right) \quad \textbf{(S26)}$$

where $A(|\theta|)$ is the free energy, k_B is the Boltzmann constant, $p(|\theta|)$ is the observed probability density for the DNA adopting a bend angle $|\theta|$, and $|\theta_0|$ is the reference bend angle, for which $A(|\theta_0|)$ = 0.

FRET efficiencies were calculated from the molecular dynamics trajectories, by adapting the accessible volume (AV) model for dye positions detailed above. Briefly, a grid of points was produced around the DNA base attached to the dye, with the spacing between grid points set to half the smallest dye dimension (see table above). Points were excluded if their distance to a base or backbone site, was smaller than the sum of the dye radius and the excluded volume radius of the base or backbone, respectively. This overlap check was repeated with the three different dye radii and the resulting AV clouds were combined (Fig S5G). A position that could accommodate all three dye radii was therefore weighted three times more than a position that could only accommodate one. The FRET efficiencies were averaged over all dye distances for each configuration and then again over all configurations in our molecular dynamics trajectories (of length ~15μs).

Single-molecule FRET measurements of DNAs in living bacteria. Gapped and duplex DNAs were internalized into electro-competent DH5α *E. coli* cells (Invitrogen) using electroporation (35). Cells were diluted 1:1 with sterile milli-Q water and stored at -80°C. For each electroporation experiment, 20 μL of electrocompetent cells were used. DNAs were stored in 2μM stocks in low-salt annealing buffer at -20 °C. For each experiment 0.25 μL of DNA and 0.2 μL of 50 mM EDTA were added to 20 μL electrocompetent cells and incubated on ice. The mixture of electro-competent cells and labeled DNAs was transferred into a pre-chilled electroporation cuvette (0.1 cm gap cuvette, Bio-Rad) and placed into an electroporator (MicroPulser, Bio-Rad). An electric field of 1.4 kV/cm was applied for electroporation. About 500 μL of super optimal broth with catabolite repression (SOC) was added immediately after electroporation. Cells were recovered for 3 min at 37°C. After recovery, cells were harvested by centrifugation at 3300 g for 1 min at 4°C and washed 5 times with 500 μL phosphate buffered saline (PBS). Cells were resuspended in 150 μL PBS and placed on 1% agarose pads before imaging. The agarose pads were made from ~300 μL of M9 medium containing 1% (v:w) BioRad Certified Molecular Biology Agarose on a coverslip. About 3 μL of cells were pipetted onto the agarose pad, and another coverslip was added on top. The slide/agar/slide sandwich was inverted and placed on the microscope with the side containing the cells closest to the objective.

Live-cell imaging was performed on a customized inverted Olympus IX-71 microscope equipped with a 532 nm DPSS laser (MGL_III-532-100mW, CNI). Laser light was collected into a single-mode optical fiber (Thorlabs, Newton, NJ, USA) and collimated before focusing on the objective. Cells were imaged using highly inclined thin illumination (HILO, Tokunaga et al., 2008) by adjusting the position of the focused excitation light on the back focal plane of the objective. Cellular fluorescence was collected through the same objective, filtered to remove excitation light through a long-pass filter (HQ545LP, Chroma) and a notch filter (NF02-633S, Semrock), and spectrally separated by a dichroic mirror (630DRLP, Omega). Donor and FRET channels were imaged onto separate halves of the chip of an electron-multiplying charge-coupled device camera (iXon+, BI-887, Andor). The illumination for brightfield images comprised a white-light lamp (IX2- ILL100, Olympus) and condenser (IX2-LWUCD, Olympus) attached to the microscope. Movies and images were recorded using manufacturer's software. Measurements were performed in green continuous-wave mode using an excitation power density of 38 W/cm^2 and 20 ms exposure time.

Custom-written MATLAB software was used to analyze single-molecule tracking and diffusion in live *E. coli* as previously described (35, 36). Briefly, the PSFs in donor and FRET channels in each movie frame were fitted by a 2D elliptical Gaussian (free fit parameters: x/y position, x/y width, elliptical rotation angle, amplitude, background) using initial position guesses from applying a fixed localization-intensity threshold on the bandpass-filtered fluorescence image (37). Tracking was performed in the FRET channel by adapting the MATLAB script based on a published algorithm (38). Localized PSFs were linked to a trajectory if they appeared in consecutive frames within a window of 7 pixels (~ 0.69 µm). This window size ensures 98% of steps are correctly linked for an apparent diffusion coefficient of 1.0 $\mu m^2/s$ at 20 ms exposure time. To account for transient disappearance of the PSF within a trajectory due to blinking or missed localization, we used a memory parameter of 1 frame. To eliminate noise, only molecules appearing in 5 consecutive frames were included in the analysis. The donor channel was mapped onto the FRET channel using a transformation matrix. FRET values, E*, were obtained from co-localized PSFs by calculating the ratio of photon counts in the FRET channel over the sum of photon counts in both channels for each single-molecule (39); E* =

$pc_{FRET}/(pc_{FRET}+pc_{Donor})$, $pc_{FRET/Donor}$: photon counts in FRET and donor channel, respectively.

Fluorescence overlay images were obtained by overlaying the donor and FRET fluorescence channels colored green and red, respectively. The green fluorescence channel was transformed onto the red fluorescence channel. All transformation matrices were based on a calibration matrix generated each day where fluorescent beads were mapped from the green onto the red fluorescence channel.

Quenchable FRET (quFRET) experiments. quFRET experiments were performed as per our standard smFRET confocal experiments (see above). DD, DA and AA photon streams were recorded and used to calculate the uncorrected FRET efficiencies (E^* - equation S9) and stoichiometries (S^* - equation S10) of filtered bursts, which correspond to individual molecules. These values were plotted as a two-dimensional histogram, and the number of bursts in the mid-S ($0.4 < S^* < 0.8$) and low-S ($S^* < 0.4$) regimes counted. One dimensional histograms of E^* were produced from projections of the mid-S data onto the E^* axis. The quFRET assay offers two related readouts for DNA melting: an increase in the absolute number of mid-S bursts, and an increase in the relative proportion of mid-S bursts compared with low-S bursts; the latter is a more robust measure, being independent of sample concentration and measurement time.

References

1. Santoso,Y., Joyce,C.M., Potapova,O., Le Reste,L., Hohlbein,J., Torella,J.P., Grindley,N.D.F. and Kapanidis,A.N. (2010) Conformational transitions in DNA polymerase I revealed by single-molecule FRET. *Proceedings of the National Academy of Sciences of the United States of America*, **107**, 715–20.

2. Kapanidis,A.N., Lee,N.K., Laurence,T. a, Doose,S., Margeat,E. and Weiss,S. (2004) Fluorescence-aided molecule sorting: analysis of structure and interactions by alternating-laser excitation of single molecules. *Proceedings of the National Academy of Sciences of the United States of America*, **101**, 8936–41.

3. Lee,N.K., Kapanidis,A.N., Wang,Y., Michalet,X., Mukhopadhyay,J., Ebright,R.H. and Weiss,S. (2005) Accurate FRET measurements within single diffusing biomolecules using alternating-laser excitation. *Biophysical journal*, **88**, 2939–53.

4. Hohlbein,J., Craggs,T.D. and Cordes,T. (2014) Alternating-laser excitation: single-molecule FRET and beyond. *Chemical Society reviews*, **43**, 1156–71.

5. Stryer,L. (1978) Fluorescence Energy Transfer as a Spectroscopic Ruler. *Ann. Rev. Biochem.*, **47**, 819–846.

6. Clegg,R.M. (1992) Fluorescence resonance energy transfer and nucleic acids. *Methods in enzymology*, **211**, 353–388.

7. Lakowicz,J.R. (2006) Principles of Fluorescence Spectroscopy Lakowicz,J.R. (ed) Springer US, Boston, MA.

8. Würth,C., Grabolle,M., Pauli,J., Spieles,M. and Resch-genger,U. (2013) Relative and absolute determination of fluorescence quantum yields of transparent samples. *Nature protocols*, **8**, 1535–50.

9. Magde,D., Wong,R. and Seybold,P.G. (2002) Fluorescence Quantum Yields and Their Relation to Lifetimes of Rhodamine 6G and Fluorescein in Nine Solvents: Improved Absolute Standards for Quantum Yields. *Photochemistry and Photobiology*, **75**, 327–334.

10. Sindbert,S., Kalinin,S., Nguyen,H., Kienzler,A., Clima,L., Bannwarth,W., Appel,B., Müller,S. and Seidel,C. a M. (2011) Accurate distance determination of nucleic acids via Förster resonance energy transfer: implications of dye linker length and rigidity. *Journal of the American Chemical Society*, **133**, 2463–80.

11. Kalinin,S., Peulen,T., Sinbert,S., Rothwell,P.J., Berger,S., Restle,T., Goody,R.S., Gohlke,H. and Seidel,C.A.M. (2012) A toolkit and benchmark study for FRET-restrained high-precision structural modeling. *Nature Methods*, **9**, 1218–25.

12. van Dijk,M. and Bonvin,A.M.J.J. (2009) 3D-DART: A DNA structure modelling server. *Nucleic Acids Research*, **37**.

13. Kiefer,J.R., Mao,C., Braman,J.C. and Beese,L.S. (1998) Visualizing DNA replication in a catalytically active Bacillus DNA polymerase crystal. *Nature*, **391**, 304–7.

14. Chinea,G., Padron,G., Hooft,R.W.W., Sander,C. and Vriend,G. (1995) The Use of Position-Specific Rotamers in Model-Building by Homology. *Proteins-Structure Function and Genetics*, **23**, 415–421.

15. Vriend,G. (1990) WHAT IF: A molecular modeling and drug design program. *Journal of Molecular Graphics*, **8**, 52–56.

16. Hornak,V., Abel,R., Okur,A., Strockbine,B., Roitberg,A. and Simmerling,C. (2006) Comparison of multiple amber force fields and development of improved protein backbone parameters. *Proteins: Structure, Function and Genetics*, **65**, 712–725.

17. Pérez,A., Marchán,I., Svozil,D., Sponer,J., Cheatham,T.E., Laughton,C. a and Orozco,M. (2007) Refinement of the AMBER force field for nucleic acids: improving the description of alpha/gamma conformers. *Biophysical journal*, **92**, 3817–29.

18. Guy,A.T., Piggot,T.J. and Khalid,S. (2012) Single-stranded DNA within nanopores: Conformational dynamics and implications for sequencing; A molecular dynamics simulation study. *Biophysical Journal*, **103**, 1028–1036.

19. Lindorff-Larsen,K., Piana,S., Palmo,K., Maragakis,P., Klepeis,J.L., Dror,R.O. and Shaw,D.E. (2010) Improved side-chain torsion potentials for the Amber ff99SB protein force field. *Proteins: Structure, Function and Bioinformatics*, **78**, 1950–1958.

20. Hess,B., Kutzner,C., Van Der Spoel,D. and Lindahl,E. (2008) GRGMACS 4: Algorithms for highly efficient, load-balanced, and scalable molecular simulation. *Journal of Chemical Theory and Computation*, **4**, 435–447.

21. Berendsen,H.J.C., Postma,J.P.M., van Gunsteren,W.F., DiNola, a and Haak,J.R. (1984) Molecular dynamics with coupling to an external bath. *The Journal of Chemical Physics*, **81**, 3684–3690.

22. Bussi,G., Donadio,D. and Parrinello,M. (2007) Canonical sampling through velocity rescaling. *The Journal of Chemical Physics*, **126**, 014101.

23. Parrinello,M. (1981) Polymorphic transitions in single crystals: A new molecular dynamics method. *Journal of Applied Physics*, **52**, 7182.

24. Darden,T., York,D. and Pedersen,L. (1993) Particle mesh Ewald: An N·log(N) method for Ewald sums in large systems. *The Journal of Chemical Physics*, **98**, 10089.

25. Humphrey,W., Dalke,A. and Schulten,K. (1996) VMD: Visual molecular dynamics. *Journal of Molecular Graphics*, **14**, 33–38.

26. Ouldridge,T.E., Louis,A.A. and Doye,J.P.K. (2011) Structural, mechanical, and thermodynamic properties of a coarse-grained DNA model. *Journal of Chemical Physics*, **134**, 0–22.

27. Šulc,P., Romano,F., Ouldridge,T.E., Rovigatti,L., Doye,J.P.K. and Louis,A.A. (2012) Sequence-dependent thermodynamics of a coarse-grained DNA model. *Journal of Chemical Physics*, **137**.

28. Romano,F., Chakraborty,D., Doye,J.P.K., Ouldridge,T.E. and Louis,A.A. (2013) Coarse-grained simulations of DNA overstretching. *Journal of Chemical Physics*, **138**, 0–10.

29. Srinivas,N., Ouldridge,T.E., Šulc,P., Schaeffer,J.M., Yurke,B., Louis,A.A., Doye,J.P.K. and Winfree,E. (2013) On the biophysics and kinetics of toehold-mediated DNA strand displacement. *Nucleic Acids Research*, **41**, 10641–10658.

30. Mosayebi,M., Romano,F., Ouldridge,T.E., Louis,A.A. and Doye,J.P.K. (2014) The role of loop stacking in the dynamics of DNA hairpin formation. *Journal of Physical Chemistry B*, **118**, 14326–14335.

31. Ouldridge,T.E., Šulc,P., Romano,F., Doye,J.P.K. and Louis,A.A. (2013) DNA hybridization kinetics: Zippering, internal displacement and sequence dependence. *Nucleic Acids Research*, **41**, 8886–8895.

32. Ahsen,N. von, Wittwer,C.T. and Schütz,E. (2001) Clinical Chemistry. *Clin. Chem.*, **39**, 804–809.

33. Russo,J., Tartaglia,P. and Sciortino,F. (2009) Reversible gels of patchy particles: Role of the valence. *Journal of Chemical Physics*, **131**.

34. Schreck,J.S., Ouldridge,T.E., Romano,F., Louis,A.A. and Doye,J.P.K. (2015) Characterizing the bending and flexibility induced by bulges in DNA duplexes. *Journal of Chemical Physics*, **142**.

35. Crawford,R., Torella,J.P., Aigrain,L., Plochowietz,A., Gryte,K., Uphoff,S. and Kapanidis,A.N. (2013) Long-lived intracellular single-molecule fluorescence using electroporated molecules. *Biophysical Journal*, **105**, 2439–2450.

36. Uphoff,S., Reyes-Lamothe,R., Garza de Leon,F., Sherratt,D.J. and Kapanidis,A.N. (2013) Single-molecule DNA repair in live bacteria. *Proceedings of the National Academy of Sciences of the United States of America*, **110**, 8063–8068.

37. Holden,S.J., Uphoff,S., Hohlbein,J., Yadin,D., Le Reste,L., Britton,O.J. and Kapanidis,A.N. (2010) Defining the limits of single-molecule FRET resolution in TIRF microscopy. *Biophysical Journal*, **99**, 3102–11.

38. Crocker,J. and Grier,D. (1996) Methods of Digital Video Microscopy for Colloidal Studies. *Journal of Colloid and Interface Science*, **179**, 298–310.

39. Plochowietz,A., El-Sagheer,A.H., Brown,T. and Kapanidis,A.. N. (2016) Stable end-sealed DNA as robust nano-rulers for in vivo single-molecule fluorescence. *Chemical Science*, **7**, 4418–4422.

Figure S1 - related to Figure 1

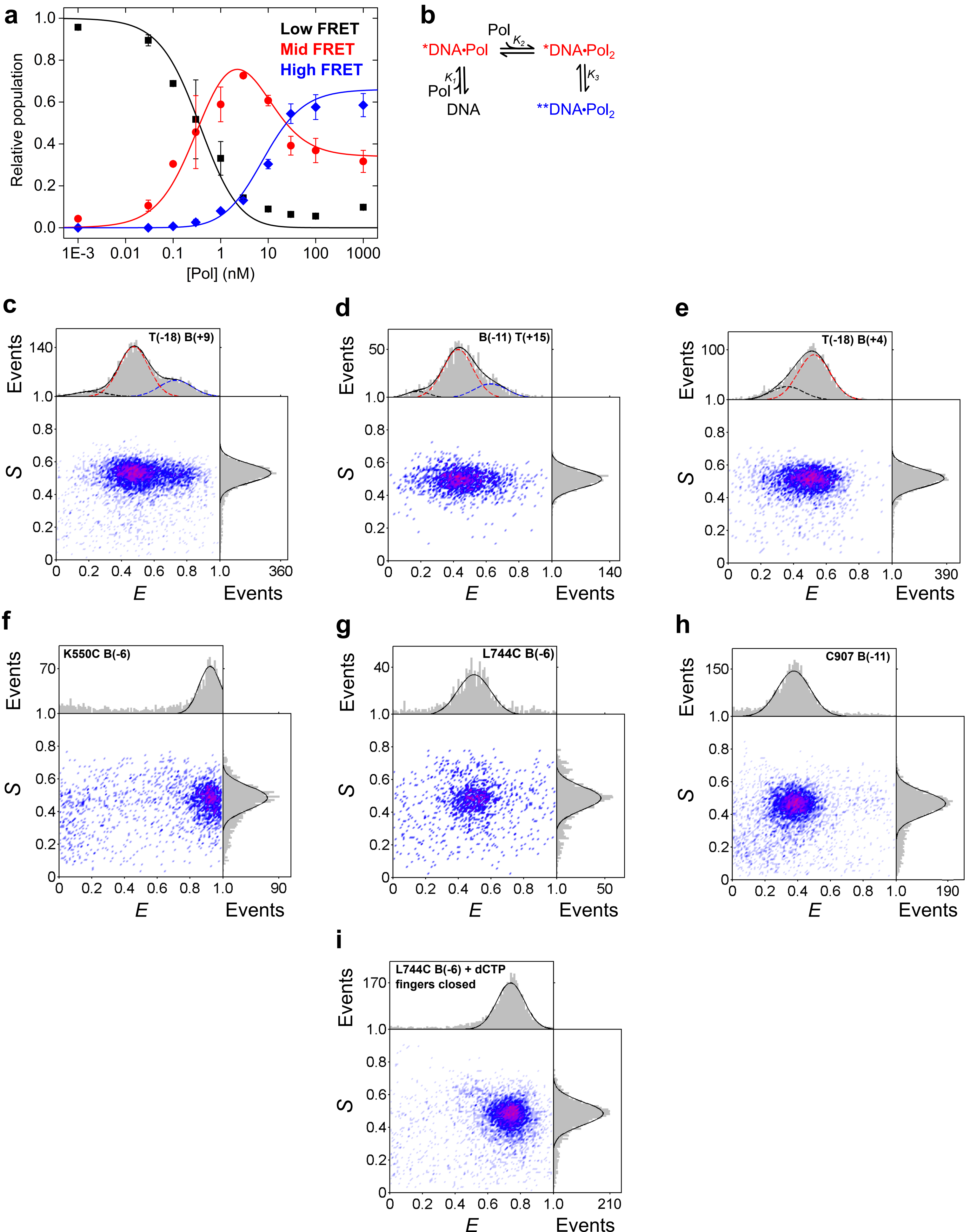

Figure S1 related to Figure 1: DNA-Pol equilibrium model and example 'ES' histograms for binary complex distance measurements

(a) Effect of Pol concentration on the relative populations of the low-FRET (black circles), mid-FRET (red circles) and high-FRET (blue circles) species. Data were globally fit to the model shown in panel B (solid lines – see *Methods*), yielding equilibrium constants: K_1 = 2.7 ± 0.3 nM^{-1}, K_2 = 0.04 ± 0.01 nM^{-1}, K_3 = 1.9 ± 0.3, and dissociation constants K_{D1} = 360 ± 60 nM and K_{D2} = 9 ± 4 nM.

(b) Schematic of the binding model for DNA-Pol interactions. DNA (low-FRET species) is bound by Pol to form *DNA·Pol binary complex (mid-FRET species), with equilibrium constant K_1*.* The binary complex can then interact with an addition Pol (without altering the FRET value), yielding a dimer species, *DNA·Pol_2 with equilibrium constant K_2. This dimer species can isomerize to a second dimer species, **DNA·Pol_2 (high-FRET) with equilibrium constant K_3.

(c – e) Examples of corrected ES histograms for DNA-DNA FRET measurements. The projection on the FRET axis was fitted by the sum of two (E) or three (C and D) Gaussians. The centre of the low FRET Gaussian (black dashed line) was fixed in the fitting, according to the DNA-only measurement for each DNA substrate (Fig 5 and Table S1). Note the corrected S value for these and all corrected ES histograms in this work, is consistently centred on 0.5, both in the case of different labelling positions (i.e., different 'ES' histograms), and for different FRET species within the same histogram. This indicates that all the corrections (for background, cross-talk and the different detection efficiencies and quantum yields of the dyes) have been performed correctly and consistently across the entire data set (see *Methods*).

(f-i) Examples of corrected ES histograms for DNA-protein FRET measurements, fitted by a single Gaussian. When the donor dye is attached to the fingers subdomain, differences in FRET are observed in the absence (panel G) and presence (panel I) of 1 mM complementary nucleotide (dCTP), corresponding to the open and closed conformations of the fingers subdomain, respectively.

Figure S2 - related to Figure 2

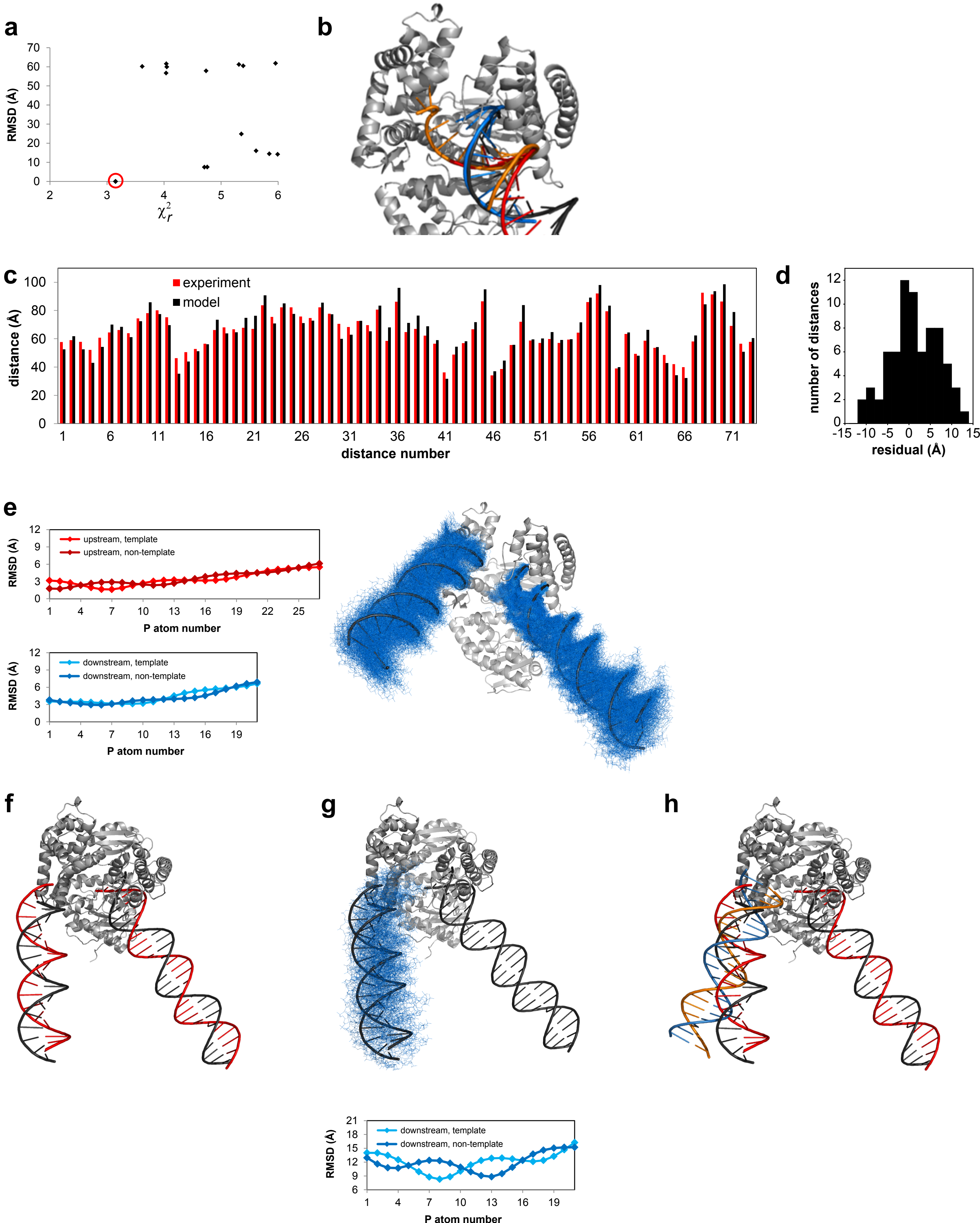

Figure S2 related to Figure 2: Rigid-body docking validation and high-FRET Pol_2-DNA ternary complex model

(A) RMSD vs reduced chi-squared plots for the refined structures (32 structures, 1 Å clash tolerance), highlighting the best model (red circle). RMSD values are calculated with comparison to the best (lowest reduced chi-squared) solution.

(B) Overlay of the upstream DNA from the crystal structure 1L3U (orange – template strand, blue –non-template strand), with the upstream DNA from our best model (red – template strand, black –non-template strand). The RMSD for equivalent phosphate atoms between the DNA in the two structures is 2.9 Å.

(C) Comparison of the 73 experimental (red) and model (black) distances obtained for the binary complex structure. See Table S1 for a list of distance numbers.

(D) Histogram of residuals from panel C.

(E) Bootstrapping analysis of the binary complex structure. The best model structure is shown with Pol in grey and DNA in black, overlaid with 100 bootstrapped structures (blue sticks). The plots show the RMSD for each DNA phosphate atom across all 100 bootstrapped structures.

(F) Best model for high-FRET Pol_2-DNA ternary structure, restrained by 21 DNA-DNA distances (Table S1). The position of the protein was modelled by aligning the upstream DNAs of the dimer and binary complex structures.

(G) Bootstrapping analysis of the high-FRET dimer structure, performed with 20 bootstrapped structures. Lower panels: RMSD for each DNA phosphate atom across all bootstrapped structures.

(H) Comparison of the position of the downstream DNA in the binary complex (orange and blue) and the high-FRET dimer structure (red and black).

Figure S3 - related to Figure 3

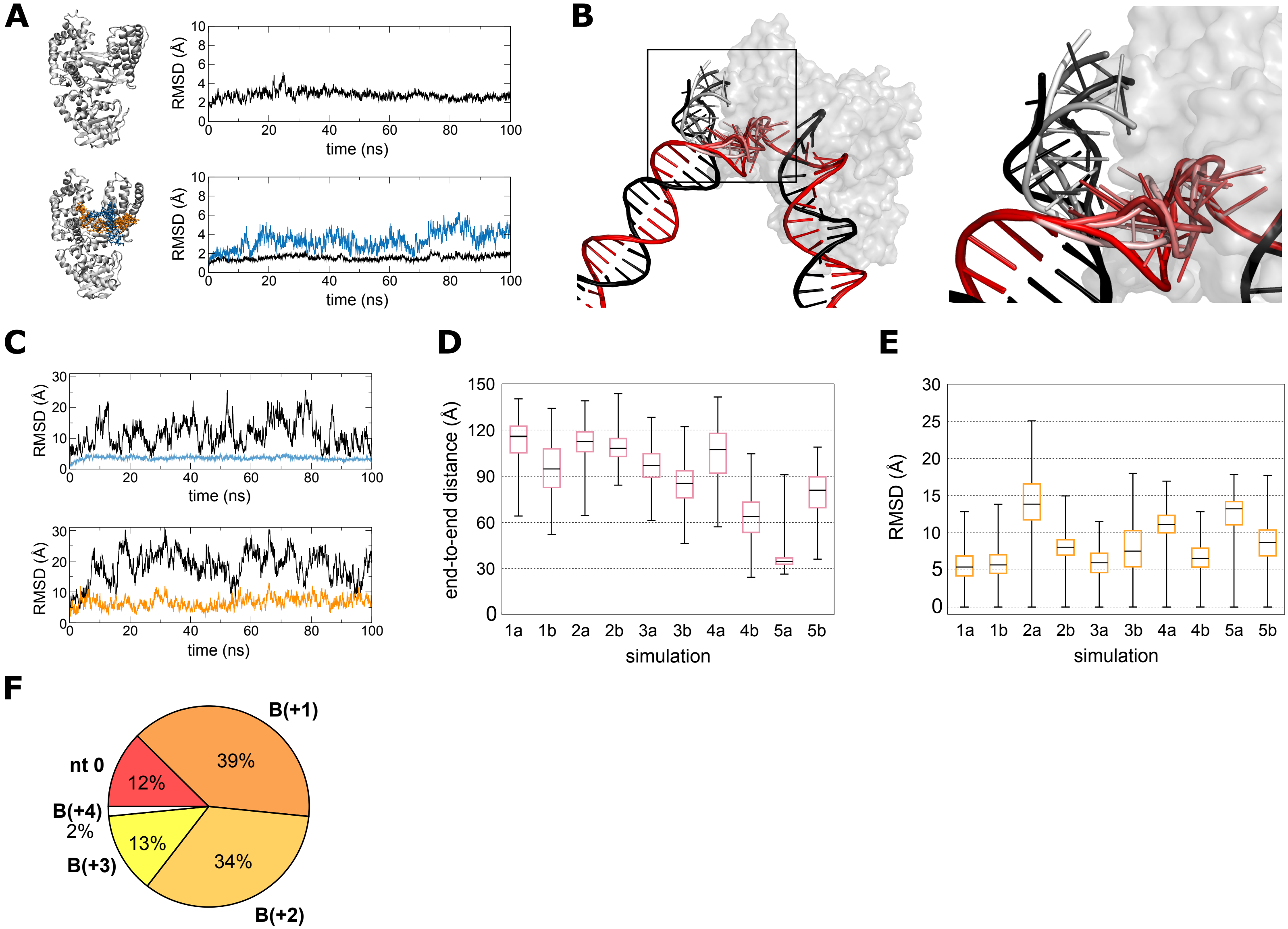

Figure S3 related to Figure 3: Molecular dynamics

(A) Control simulations of Pol 4BDP structure, in the apo state (top left) and with the X-ray DNA present (bottom left). The snapshots indicate representative conformations of the protein in the two simulations. Right panels: RMSD of C-alpha protein atoms relative to C-alpha atoms (black), and the RMSD of DNA atoms relative to C-alpha atoms (blue).

(B) An overlay of the five conformations of DNA that emerged from high-temperature simulations, which were subsequently used as starting models for the binary complex production runs. The region that was allowed to move and therefore differs between the models (shown zoomed-in on the right) is represented with different color intensities (highest, model 1; lowest, model 5). The protein is transparent for clarity.

(C) Dynamics of upstream (top) and downstream DNA (bottom), for one example simulation. The plots show the RMSD of upstream or downstream DNA atoms relative to C-alpha protein atoms (black), of the part of upstream DNA present in the X-ray structure relative to C-alpha atoms (blue), and of the 6-nt non-template flap relative to the rest of downstream DNA (orange).

(D) The range of end-to-end distances accessible to the complex in each of the ten simulations, with simulation numbers (1-5) referring to the different starting models, and letters (a or b) indicating repeats. Black lines are median values, boxes denote the 25th and 75th percentiles, and whiskers show the minimum and maximum values.

(E) The range of RMSD values for the 6-nucleotide non-template flap, relative to the rest of downstream DNA, in each of the ten simulations. The representation is as in panel D.

(F) The frequency of each of the bases of downstream residues 0-4 of the template strand being the closest residue to the side chain of the strand-separating Pol residue Y719, during the entire 1-μs simulation time.

Figure S4 - related to Figure 4

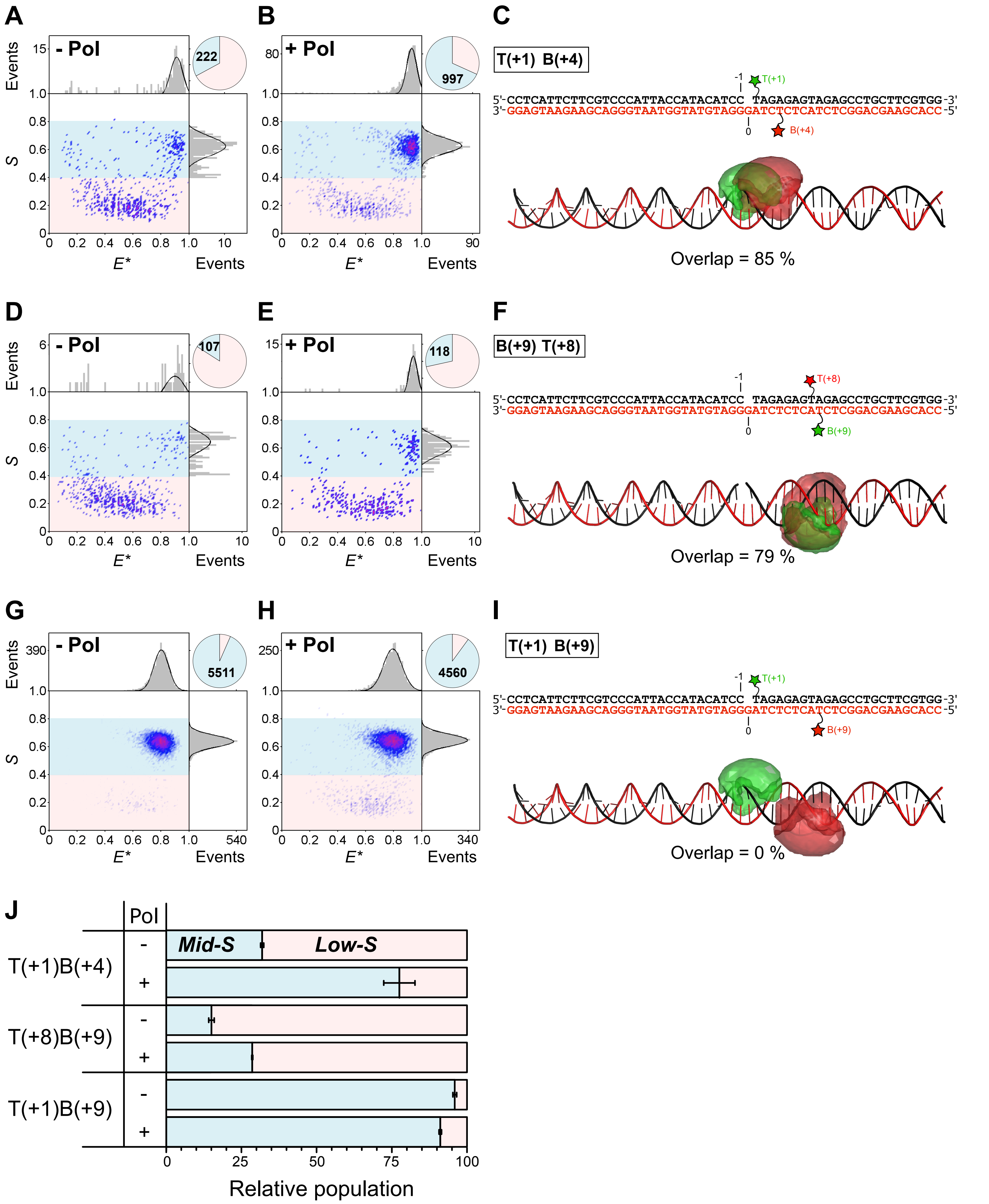

Figure S4 related to Figure 4: Quenchable FRET

(A) ES histogram for T(+1)B(+4) DNA substrate in the absence of Pol; inset pie chart shows the proportions of events with intermediate –S (0.4 < S < 0.8 – pale blue) and low-S (S< 0.4 – pale pink), quoting the number of mid-S events.

(B) As for A, but in the presence of 3 nM Pol.

(C) Schematic and AV clouds for the T(+1)B(+4) DNA substrate, quoting the percentage of the donor AV that overlaps the acceptor AV.

(D-F) as for (A-C) but for the B(+9)T(+8) gapped substrate.

(G-I) as for (A-C) but for the T(+1)B(+9) gapped substrate.

(J) Summary of Intermediate-S / Low-S percentages in the presence and absence of 3 nM Pol. Data are shown as the mean and S.E.M from two experimental repeats.

Figure S5 - related to Figure 5

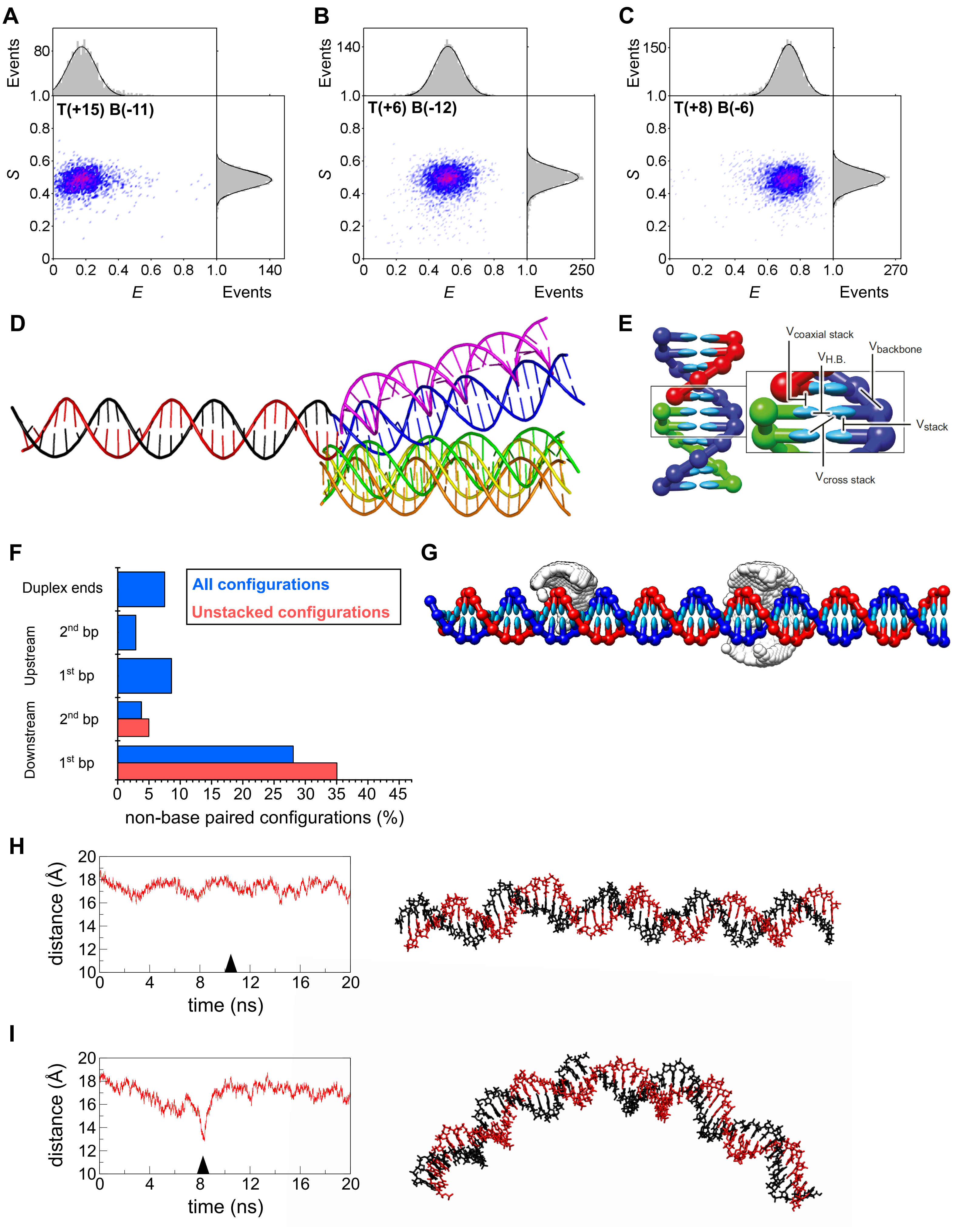

Figure S5 related to Figure 5: Free DNA substrate structure and dynamics

(A-C) ES histograms for DNA-DNA FRET measurements of the 1-nt gapped DNA substrate in the absence of Pol. (A) T(+15)B(-11), E = 0.17; (B) T(-12)B(+6) E = 0.52; (C) T(+8)B(-6), E = 0.73.

(D) Structural models of the 1nt-gapped substrate obtained from rigid-body docking calculations. The bets five models are shown with the lowest reduced chi-squared values: 2.26 (yellow), 2.26 (orange), 2.28 (pink), 2.55 (blue), and 3.54 (green).

(E) Illustration of a DNA double helix in the oxDNA model and the different interaction terms that stabilise the structure. The bases are represented by cyan ellipsoids and the backbone sites by spheres. The backbone colouring indicates the strand identity. All nucleotides also interact with repulsive excluded-volume interactions (*Methods*).

(F) Fraying propensity for base pairs immediately upstream and downstream of the 1-nt gap and at the duplex ends. Data is shown for all configurations (blue) and the subset of unstacked (red) configurations. The different fraying propensity observed immediately upstream and downstream of the gap arises from the different base pairs present at these positions (CG – upstream vs TA – downstream). A base pairing / stacking interaction is considered to be broken if its Hydrogen-bonding / stacking energy becomes larger than -0.596 kcal mol^{-1} (or -kT at T=300K).

(G) The accessible volumes (grey spheres) of donor and acceptor dyes calculated in the oxDNA model (*Methods*).

(H – I) All-atom MD of duplex (H) and 1nt gapped (I) DNA substrates. The plot shows the DNA end-to-end distance fluctuations over the simulations, with the ends taken as the terminal non-hydrogen atoms of the template strands. The arrowheads indicate the time points of the snapshots shown.

Figure S6 - related to Figure 6

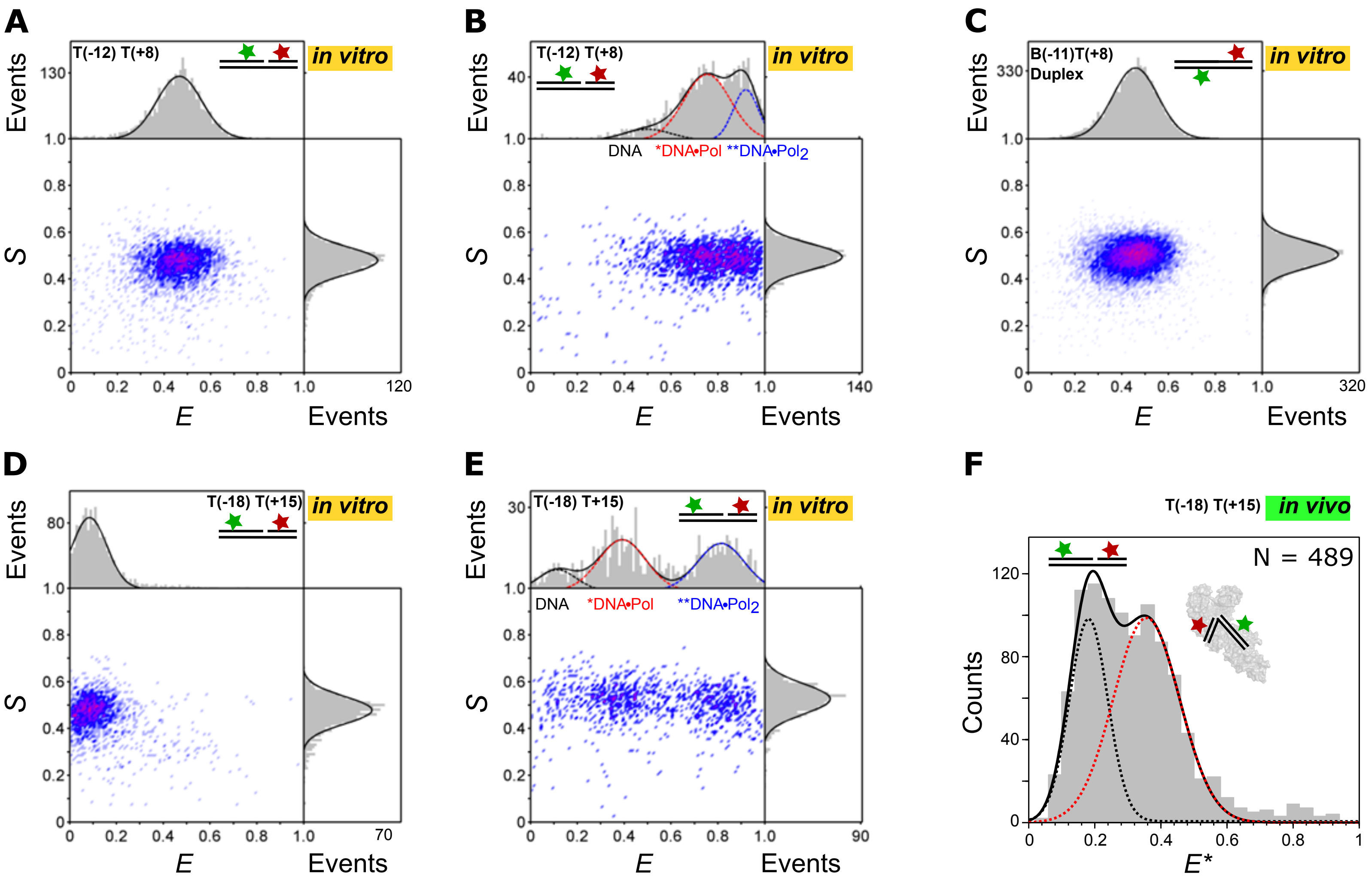

Figure S6 related to Figure 6: *In vivo* detection of bent gapped DNA

(A-C). ES histograms of *in vitro* controls for the *in vivo* gapped-DNA bending assay. (A) T(-12)T(+8) gapped DNA alone, E=0.47±0.10. (B) T(-12)T(+8) gapped DNA in the presence of 3 nM Pol showing three FRET species, assigned to the unbound DNA (E = 0.50±0.10), binary complex (E = 0.75±0.10), and the high-FRET ternary complex (E = 0.92±0.05). (C) B(-11)T(+8) *Duplex* DNA , E = 0.46±0.10.

(D-F) Dimer formation assay *in vivo* and *in vitro*. (D) *In vitro* control of T(-18)T(+15) gapped DNA alone, unbound DNA=0.08±0.07. (E) *In vitro* control of T(-18)T(+15) gapped DNA in presence of 3 nM KF(DNA Pol I) contains three FRET species: unbound DNA
(E = 0.11±0.08), binary complex, (E = 0.39±0.10), and high-FRET ternary complex (E = 0.81±0.10). (F) *In vivo* FRET histogram of T(-18)T(+15) gapped DNA showing two FRET species: unbound DNA (E = 0.19±0.07) and binary complex (E = 0.35±0.10). All E values are quoted as (mean ± s.d. of the Gaussian fits).

Table S1: Binary complex and high-FRET ternary complex distances – measured and modelled

			smFRET				R_{mp} (model) (Å)	
Distance Number	Donor	Acceptor	Mid-FRET	High-FRET	$<R_{DA}>_E$ (Å)	R_{mp} (Å)	Binary Complex	High-FRET Ternary Complex
1	T(-12)	B(+11)	0.634	0.896	58.9	57.7	52.6	44.0
2	T(-12)	B(+9)	0.604	0.768	60.1	59.1	61.8	44.9
3	T(-12)	B(+6)	0.631	-	59.0	57.9	52.5	-
4	T(-12)	B(+4)	0.744	-	54.0	52.2	43.0	-
5	T(-18)	B(+11)	0.568	0.807	61.6	60.8	54.3	51.9
6	T(-18)	B(+9)	0.488	0.731	65.0	64.5	70.1	48.0
7	T(-18)	B(+6)	0.453	-	66.6	66.2	68.5	-
8	T(-18)	B(+4)	0.501	-	64.5	63.9	61.2	-
9	T(-22)	B(+11)	0.301	0.616	74.2	74.4	72.4	62.9
10	T(-22)	B(+9)	0.244	0.428	77.9	78.1	85.8	65.4
11	T(-22)	B(+6)	0.219	-	79.7	80.1	77.5	-
12	T(-22)	B(+4)	0.287	-	75.1	75.2	69.6	-
13	B(-6)	T(+8)	0.840	-	48.9	46.3	35.3	-
14	B(-11)	T(+8)	0.774	-	52.5	50.5	43.9	-
15	T(-12)	T(+8)	0.732	0.919	54.6	52.8	51.2	41.2
16	T(-18)	T(+8)	0.659	-	57.8	56.5	56.1	-
17	T(-22)	T(+8)	0.453	-	66.6	66.2	73.5	-
18	B(-6)	T(+15)	0.414	0.574	68.3	68.1	63.8	58.2
19	B(-11)	T(+15)	0.441	0.618	67.1	66.7	64.6	52.4
20	T(-12)	T(+15)	0.419	0.663	68.1	67.8	74.8	63.0
21	T(-18)	T(+15)	0.436	0.825	67.3	67.0	76.3	56.7
22	T(-22)	T(+15)	0.178	0.372	83.2	83.7	90.7	72.4
23	T(-24)	B(+11)	0.282	0.607	75.4	75.5	70.8	60.7
24	T(-24)	B(+9)	0.194	0.368	81.7	82.2	85.0	64.6
25	T(-24)	B(+6)	0.194	-	81.8	82.2	77.6	-
26	T(-24)	B(+4)	0.278	-	75.6	75.8	71.1	-
27	T(-24)	T(+8)	0.296	0.542	74.5	74.7	72.9	62.5
28	T(-24)	T(+15)	0.194	0.478	81.8	82.2	85.6	66.1
29	T(-24)	T(+18)	0.249	0.662	77.5	77.8	77.4	53.8
30	B(-6)	T(+18)	0.366	-	70.7	70.6	60.0	63.3
31	B(-11)	T(+18)	0.409	0.721	68.6	68.3	62.9	46.1
32	T(-12)	T(+18)	0.331	0.668	72.6	72.6	72.7	58.4
33	T(-18)	T(+18)	0.383	0.601	69.8	69.7	65.3	55.3
34	T(-22)	T(+18)	0.213	0.491	80.2	80.6	83.4	62.4
35	550	T(+8)	0.486		59.5	58.5	68.1	
36	550	T(+15)	0.097		85.7	86.2	96.0	
37	550	B(+11)	0.354		65.2	64.8	71.2	
38	550	B(+9)	0.312		67.3	67.0	76.4	
39	550	B(+6)	0.406		62.9	62.2	68.9	
40	550	B(+4)	0.532		57.8	56.5	59.1	
41	550	T(-12)	0.901		40.8	36.3	31.7	
42	550	T(-18)	0.704		51.1	48.8	54.4	
43	550	T(-22)	0.522		58.1	56.9	58.3	
44	550	T(-24)	0.318		67.0	66.7	71.8	
45	550	T(+18)	0.095		86.0	86.5	95.0	
46	550	B(-6)	0.922		39.1	34.1	37.0	
47	550	B(-11)	0.875		42.7	38.6	44.6	
48	907	T(+8)	0.552		57.0	55.6	55.7	
49	907	T(+15)	0.233		72.0	72.0	83.9	
50	907	B(+11)	0.478		59.9	58.8	59.6	
51	907	B(+9)	0.517		58.3	57.1	60.2	
52	907	B(+6)	0.455		60.8	59.9	64.7	
53	907	B(+4)	0.519		58.2	57.0	59.2	
54	907	T(-12)	0.467		60.3	59.4	59.7	
55	907	T(-18)	0.363		64.8	64.3	71.6	
56	907	T(-22)	0.098		85.4	86.0	89.1	
57	907	T(-24)	0.068		91.3	92.1	98.0	
58	907	T(+18)	0.147		79.1	79.4	83.4	
59	907	B(-6)	0.870		43.0	39.0	40.0	
60	907	B(-11)	0.381		64.0	63.4	64.5	
61	744	T(+8)	0.692		51.6	49.4	48.1	
62	744	T(+15)	0.481		59.8	58.7	66.4	
63	744	B(+11)	0.599		55.2	53.6	54.1	
64	744	B(+9)	0.707		50.9	48.6	42.9	
65	744	B(+6)	0.826		45.5	42.1	34.3	
66	744	B(+4)	0.858		43.7	39.9	32.3	
67	744	T(-12)	0.494		59.2	58.1	62.3	
68	744	T(-18)	0.065		91.9	92.6	84.4	
69	744	T(-22)	0.070		90.7	91.4	93.6	
70	744	T(-24)	0.096		85.8	86.3	98.5	
71	744	T(+18)	0.276		69.3	69.1	78.9	
72	744	B(-6)	0.532		57.7	56.5	50.8	
73	744	B(-11)	0.500		59.0	57.9	60.5	

Note: The standard errors on all smFRET measurements are estimated as ± 0.05 *E* based on three repeats of an example measurement.

Table S2 – related to Figure 5: DNA substrates alone - measured and modelled FRET

Labeling index	Donor	Acceptor	smFRET[a]	oxDNA – FRET[b] Gap	Nick	Duplex	Docked gap[c]	B-DNA gap[d]
1	B(-11)	T(+8)	0.502	0.504	0.443	0.439	0.563	0.466
2	T(-22)	T(+8)	0.103	0.110	0.078	0.077	0.098	0.070
3	T(-12)	B(+11)	0.369	0.351	0.291	0.290	0.412	0.258
4	T(-12)	B(+9)	0.407	0.461	0.374	0.366	0.600	0.359
5	T(-12)	B(+6)	0.513	0.511	0.470	0.471	0.621	0.445
6	T(-12)	B(+4)	0.649	0.632	0.635	0.647	0.690	0.609
7	T(-18)	B(+11)	0.128	0.118	0.078	0.077	0.156	0.069
8	T(-18)	B(+9)	0.167	0.157	0.112	0.111	0.203	0.092
9	T(-18)	B(+6)	0.240	0.238	0.236	0.237	0.183	0.199
10	T(-18)	B(+4)	0.347	0.334	0.332	0.334	0.281	0.323
11	T(-22)	B(+11)	0.112	0.079	0.051	0.050	0.068	0.040
12	T(-22)	B(+9)	0.113	0.102	0.065	0.063	0.104	0.055
13	T(-22)	B(+6)	0.142	0.112	0.090	0.090	0.123	0.078
14	T(-22)	B(+4)	0.189	0.147	0.138	0.142	0.170	0.118
15	B(-6)	T(+8)	0.726	0.711	0.658	0.653	0.838	0.698
16	T(-12)	T(+8)	0.459	0.506	0.444	0.440	0.548	0.457
17	T(-18)	T(+8)	0.196	0.168	0.121	0.119	0.224	0.116
18	B(-6)	T(+15)	0.348	0.283	0.273	0.276	0.326	0.276
19	B(-11)	T(+15)	0.168	0.181	0.118	0.114	0.152	0.123
20	T(-12)	T(+15)	0.186	0.163	0.106	0.104	0.167	0.103
21	T(-18)	T(+15)	0.074	0.068	0.044	0.043	0.048	0.036
22	T(-22)	T(+15)	0.022	0.046	0.022	0.021	0.028	0.019
23	T(-24)	B(+11)	0.073	0.060	0.035	0.035	0.053	0.026
24	T(-24)	B(+9)	0.054	0.082	0.052	0.051	0.072	0.040
25	T(-24)	B(+6)	0.072	0.094	0.071	0.069	0.094	0.062
26	T(-24)	B(+4)	0.104	0.108	0.090	0.090	0.148	0.079
27	T(-24)	T(+8)	0.078	0.081	0.055	0.055	0.075	0.045
28	T(-24)	T(+15)	0.025	0.042	0.018	0.018	0.021	0.015
29	T(-24)	T(+18)	0.008	0.037	0.013	0.013	0.016	0.010
30	B(-6)	T(+18)	0.216	0.188	0.150	0.149	0.260	0.151
31	B(-11)	T(+18)	0.119	0.132	0.083	0.081	0.096	0.073
32	T(-12)	T(+18)	0.105	0.129	0.081	0.080	0.098	0.070
33	T(-18)	T(+18)	0.057	0.052	0.025	0.025	0.039	0.021
34	T(-22)	T(+18)	0.088	0.041	0.017	0.017	0.020	0.013
**Mean Deviation[e]**				**-0.0025**	**-0.037**	**0.038**	**0.012**	**-0.045**
**RMSD[f]**				**0.027**	**0.045**	**0.046**	**0.054**	**0.052**

a) Experimentally measured and corrected FRET value for each donor-acceptor pair: Standard error = ± 0.05 *E*
b) FRET values calculated for each donor-acceptor pair, averaged over the entire coarse-grained simulations for the 1-nt gapped (gap), nick and duplex substrates.
c) FRET values arising from a static gapped structure generated by rigid-body docking (see Fig S5).
d) FRET values arising from a static-gapped structure generated by removing one nucleotide from a B-form DNA duplex.
e) Mean deviation = $<E_{model} – E_{measured}>$
f) RMSD = SQRT($<(E_{model} – E_{measured})^2>$)

Table S3: Oligonucleotide sequences for labelled DNA substrates

	Name	Sequence
[a]DNA1	T(-NL)	5′-CCTCATTCTTCGTCCCATTACCATACATCC$_H$-3′
	T(-24)	5′-CCTCATXCTTCGTCCCATTACCATACATCC$_H$-3′
	T(-22)	5′-CCTCATTCXTCGTCCCATTACCATACATCC$_H$-3′
	T(-18)	5′-CCTCATTCTTCGXCCCATTACCATACATCC$_H$-3′
	T (-12)	5′-CCTCATTCTTCGTCCCATXACCATACATCC$_H$-3′
[b]DNA2	T(+NL)	5′-TAGAGAGTAGAGCCTCGTTCGTGG-3′
	T(+1)	5′-XAGAGAGTAGAGCCTCGTTCGTGG-3′
	T(+8)	5′-TAGAGAGXAGAGCCTCGTTCGTGG-3′
	T(+15)	5′-TAGAGAGTAGAGCCXCGTTCGTGG-3′
	T(+18)	5′-TAGAGAGTAGAGCCTCGXTCGTGG-3′
DNA3	B(NL)	5′- CCACGAACGAGGCTCTACTCTCTAGGGATGTATGGTAATGGGACGAAGAATGAGG-3′
	B(-11)	5′- CCACGAACGAGGCTCTACTCTCTAGGGATGTATGGXAATGGGACGAAGAATGAGG-3′
	B(-6)	5′- CCACGAACGAGGCTCTACTCTCTAGGGATGXATGGTAATGGGACGAAGAATGAGG-3′
	B(+4)	5′- CCACGAACGAGGCTCTACTCXCTAGGGATGTATGGTAATGGGACGAAGAATGAGG-3′
	B(+6)	5′- CCACGAACGAGGCTCTACXCTCTAGGGATGTATGGTAATGGGACGAAGAATGAGG-3′
	B(+9)	5′- CCACGAACGAGGCTCXACTCTCTAGGGATGTATGGTAATGGGACGAAGAATGAGG-3′
	B(+11)	5′- CCACGAACGAGGCXCTACTCTCTAGGGATGTATGGTAATGGGACGAAGAATGAGG-3′
[c]Duplex	TD(+8)	5′-CCTCATTCTTCGTCCCATTACCATACATCCCTAGAGAGXAGAGCCTCGTTCGTGG-3′

X – indicated the position of a dT – C6 – NH_2 group used for dye labelling.

a) DNA1 oligos contain a 5’ –biotin and terminate with a dideoxy C nucleotide, denoted C_H.

b) DNA2 sequences start with a 5’ phosphorylated nucleotide.

c) Used to make the B(-11)T(+8) duplex used in the *in vivo* experiments.

Table S4: R_0 determination and anisotropy measurements

Cy3B attachment	Atto647N attachment	Quantum Yield (Cy3B)	Overlap integral J (M^{-1} cm^{-1} nm^3)	Förster Radius R_0 (Å)	Anisotropy (Cy3B)	Anisotropy (Atto647N)
free dye	free dye	0.644	4.88E+15	**62.3**	0.056	0.048
DNA	DNA	0.811	4.90E+15	**64.8**	0.185	0.159
DNA (+ Pol)[a]	DNA (+ Pol)[a]	0.794	4.81E+15	**64.4**	0.206	0.201
KF-550	DNA	0.436	4.98E+15	**58.6**	0.214	0.201
KF-744	DNA	0.421	4.91E+15	**58.1**	0.224	0.201
KF-907	DNA	0.486	4.91E+15	**59.5**	0.265	0.201

a) Measurements taken in the presence of 1 nM Pol.
